# Lipidated apolipoprotein E4 structure and its receptor binding mechanism determined by a combined cross-linking coupled to mass spectrometry and molecular dynamics approach

**DOI:** 10.1371/journal.pcbi.1006165

**Published:** 2018-06-22

**Authors:** Nicolas Henry, Eva-Maria Krammer, Florian Stengel, Quentin Adams, François Van Liefferinge, Ellen Hubin, Rui Chaves, Rouslan Efremov, Ruedi Aebersold, Guy Vandenbussche, Martine Prévost, Vincent Raussens, Stéphanie Deroo

**Affiliations:** 1 Center for Structural Biology and Bioinformatics, Structure and Function of Biological Membranes, Faculté des Sciences, Université Libre de Bruxelles (ULB), Brussels, Belgium; 2 Department of Biology, Institute of Molecular Systems Biology, ETH Zurich, Zurich, Switzerland; 3 Department of Biology, University of Konstanz, Konstanz, Germany; 4 Structural Biology Research Center, VIB, Brussels, Belgium; 5 Structural Biology Brussels, Department of Biotechnology (DBIT), Vrije Universiteit Brussel (VUB), Brussels, Belgium; 6 Nanobiophysics Group, MIRA Institute for Biomedical Technology and Technical Medicine, Faculty of Science and Technology, University of Twente, Enschede, The Netherlands; 7 Faculty of Science, University of Zurich, Zurich, Switzerland; University of Houston, UNITED STATES

## Abstract

Apolipoprotein E (apoE) is a forefront actor in the transport of lipids and the maintenance of cholesterol homeostasis, and is also strongly implicated in Alzheimer’s disease. Upon lipid-binding apoE adopts a conformational state that mediates the receptor-induced internalization of lipoproteins. Due to its inherent structural dynamics and the presence of lipids, the structure of the biologically active apoE remains so far poorly described. To address this issue, we developed an innovative hybrid method combining experimental data with molecular modeling and dynamics to generate comprehensive models of the lipidated apoE4 isoform. Chemical cross-linking combined with mass spectrometry provided distance restraints, characterizing the three-dimensional organization of apoE4 molecules at the surface of lipidic nanoparticles. The ensemble of spatial restraints was then rationalized in an original molecular modeling approach to generate monomeric models of apoE4 that advocated the existence of two alternative conformations. These two models point towards an activation mechanism of apoE4 relying on a regulation of the accessibility of its receptor binding region. Further, molecular dynamics simulations of the dimerized and lipidated apoE4 monomeric conformations revealed an elongation of the apoE N-terminal domain, whereby helix 4 is rearranged, together with Arg172, into a proper orientation essential for lipoprotein receptor association. Overall, our results show how apoE4 adapts its conformation for the recognition of the low density lipoprotein receptor and we propose a novel mechanism of activation for apoE4 that is based on accessibility and remodeling of the receptor binding region.

## Author summary

Among the proteins involved in the transport of lipids and their distribution to the cells, apolipoprotein E (apoE) mediates the internalization of cholesterol rich lipoproteins by acting as a ligand for cell-surface receptors. In the central nervous system, while apoE is the major cholesterol transport protein, a dysfunction of apoE in the transport and metabolism of lipids is associated with Alzheimer’s disease. A molecular understanding of the mechanisms underlying the receptor binding abilities of apoE is crucial to address its biological functions, but is so far hindered by the dynamic and complex nature of these assemblies. We have designed an original hybrid approach combining experimental data and bioinformatics tools to generate high resolution models of lipidated apoE. Based on these models, we can propose how apoE adapts its conformation at the surface of lipid nanoparticles. Further, we propose a novel mechanism of regulation of the activation and receptor recognition of apoE that could prove valuable to interpret its role in Alzheimer and apoE-related cardiovascular diseases.

## Introduction

Apolipoprotein E (apoE) is a member of the superfamily of exchangeable apolipoproteins. It mediates cellular uptake of cholesterol-rich lipoproteins by acting as a high affinity ligand for cell surface receptors belonging to the low-density lipoprotein (LDL) receptor family [1]. An imbalance in cholesterol homeostasis increases the risk for cardiovascular diseases and is also linked to neurodegenerative disorders [2,3]. Therefore, the receptor binding property of apoE stresses its importance in the transport of lipids and metabolism of cholesterol both within the plasma and the central nervous system [4,5]. In blood plasma, the receptor mediated uptake and endocytosis of apoE-containing lipoproteins lowers the overall levels of circulating lipoproteins, explaining the anti-atherogenic effect of apoE [6]. In the brain, although apoE is involved in lipid redistribution and neuronal growth and repair, the presence of the ε4 allelic form of the apoE gene also represents the most significant genetic risk factor of developing Alzheimer’s disease [7]. An abnormal trafficking of lipids and cholesterol by apoE4 is among the pathogenic mechanisms that are proposed to contribute to the susceptibility of ε4 carriers for Alzheimer’s disease [8,9].

ApoE is a ~34 kDa protein composed of 299 amino acids. Single point variations at positions 112 and 158 distinguish the three main isoforms of apoE: apoE2 (Cys112, Cys158), apoE3 (Cys112, Arg158) and apoE4 (Arg112, Arg158) [10]. These sole amino acid substitutions result in structural differences between these isoforms [11] and marked effects on their lipid binding abilities [12], providing grounds to explain their different physiological role(s) in cardiovascular and Alzheimer’s diseases [13]. In the lipid-free state, all three apoE isoforms possess two independently folded structural domains linked by a protease sensitive loop [14]. The N-terminal (NT) domain (res. 1 to 191) comprises an elongated four-helix bundle that contains the binding region to the members of the LDL receptor family on the fourth helix [15]. The C-terminal (CT) domain (res. 210 to 299) presents the major lipid binding region [16] and is particularly challenging to study, as it is involved in the oligomerization of apoE in the absence of lipids [17]. Several mutations had to be introduced in the CT domain to generate a stable monomeric protein leading to the so far only available full-length high resolution three-dimensional (3D) structure of a lipid-free apoE protein. In this structure, the CT domain variant contains three α-helices folded upon the NT domain conferring a globular shape to apoE [18].

Upon binding to lipid particles, apoE undergoes a large conformational conversion to accommodate and stabilize the lipids through its amphipathic α-helices, allowing thereby their trafficking in the circulation [19]. Additionally, lipid binding induces apoE to adopt a biologically active conformation that is a prerequisite for the binding of lipoproteins to cell surface LDL receptors and their internalization [1]. Analysis of reconstituted discoidal phospholipid-apoE particles (rHDL, more recently termed nanodisk) presented a major step forward towards a structure of lipid-bound apoE [19]. These particles mimic *in vivo* nascent high density lipoproteins (HDL) in shape, size, density and functional properties [20]. It was demonstrated that in these systems, the α-helices of apoE are oriented perpendicularly to the acyl chains of the lipids and the apolipoprotein molecules circumscribe the edge of the discoidal particles [21–23]. Lipid-binding also triggers the elongation of NT domain helix 4 which was proposed to represent a key lipid-induced conformational change allowing for the recognition of apoE by LDL receptors [24,25]. However, the conformation adopted by apoE molecules at the surface of these discoidal particles remains an open question. While it is accepted that the CT domain adopts an extended α-helical structure [19, 22, 23], the conformation of the NT domain has not converged towards a single model. Based on calorimetry measurements, it was proposed that the four-helix bundle opens to expose the hydrophobic faces of the amphipathic helices towards the lipids and that further reorganization of helices occurs, triggered by lipid binding [26]. Although this bundle opening was suggested to ultimately lead to a fully extended conformation of apoE that wraps around the entire circumference of the lipid bilayer of the disc [22], several studies have indicated that apoE adopts a hairpin structure for which distinct hinge localizations were proposed [27–29]. Supported by low resolution X-ray density and electron paramagnetic resonance (EPR) measurements, an alternative model was developed. In this case, even though apoE also folds in a hairpin structure, the hydrophobic faces of apoE helices are suggested to interact with each other, while the polar faces contact the phospholipids leading to ellipsoidal lipoparticles [30,31]. Despite two decades of intensive structural studies, a consensus on the conformation of lipidated apoE has not yet been reached.

**Fig 1:**
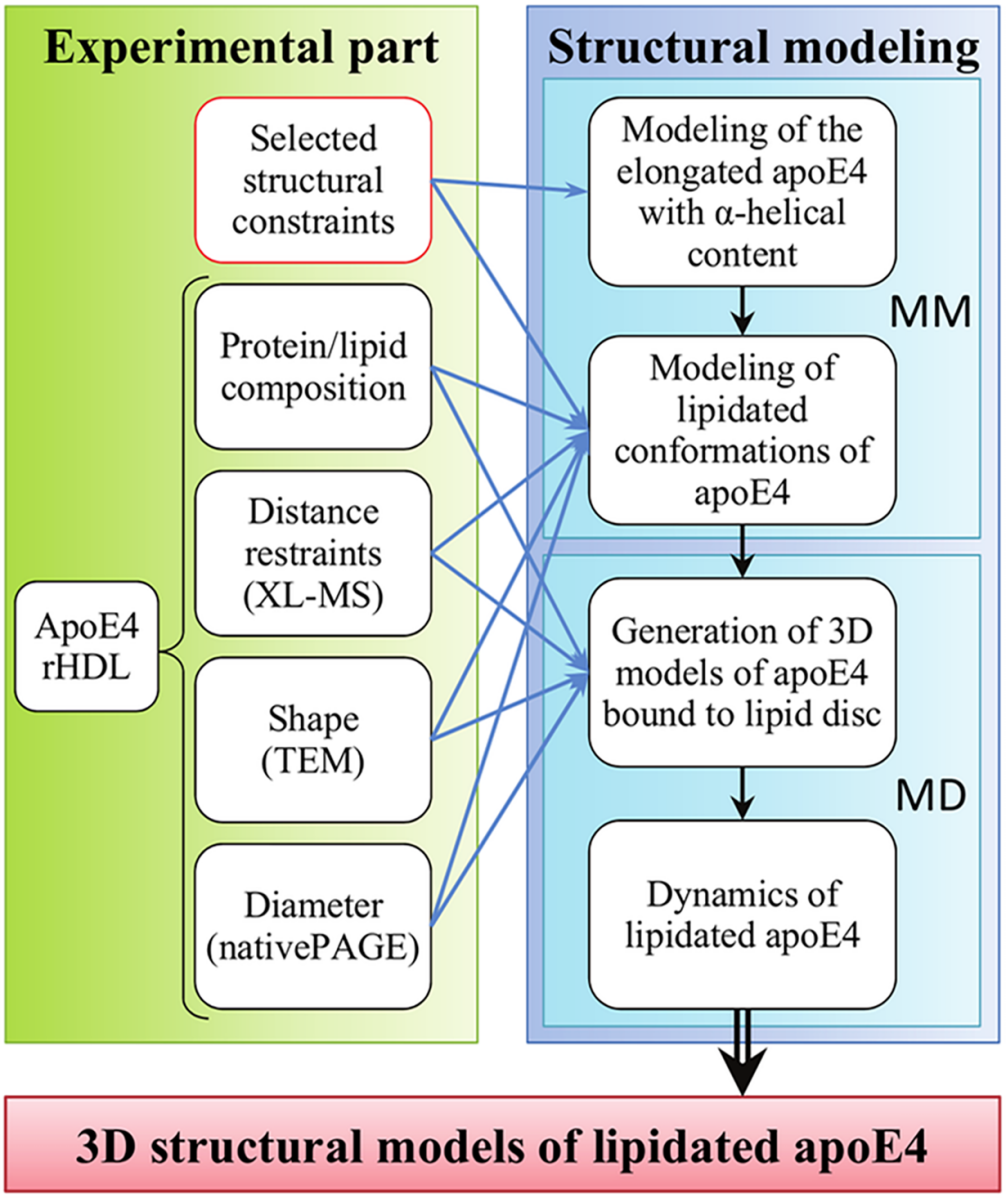
Hybrid structural method for predicting the 3D structure of apoE4 bound to rHDL. The molecular structure of lipidated apolipoprotein E4 (apoE4) is elucidated by using a combination of experimental techniques, e.g. chemical cross-linking coupled to mass spectrometry (XL-MS), transmission electron microscopy (TEM) and native gel electrophoresis (native PAGE) combined with molecular modeling (MM) and molecular dynamics (MD). Each step of this study is depicted in the black framed boxes. The red framed box indicates data coming from the literature. Blue arrows indicate data used as input for the different steps of the modeling process and black arrows the workflow of the structural modeling.

With the aim of deciphering the molecular structure adopted by apoE at the surface of rHDL in solution, we designed an approach where complementary low resolution structural data were combined with 3D structural modeling (Fig 1). We decided to focus our present work on rHDLs containing only apoE4, considering the prevalent role of this isoform in Alzheimer’s disease [8,9]. Experimental data were primarily generated from chemical cross-linking (XL) coupled to mass spectrometry (-MS) which produces covalently connected pairs of peptides that provide a set of distance restraints between cross-linked residues on the native protein, enabling low resolution models to be elaborated. XL-MS has seen significant progress recently [32–37] and has been successfully applied to a large number of protein complexes [38–41]. The distance restraints from our intramolecular XLs, together with additional experimental data obtained in this work and information from the literature were then used in our hybrid molecular modeling approach. Two alternative models of lipidated apoE4 were validated by our XL-MS results and assessed by molecular dynamics simulations. Our resulting models represent the most detailed structures obtained so far on full-length apoE4 associated to rHDL and they provide unprecedented insight into the active structure of apoE4. Taken together the data allowed us to propose a novel molecular mechanism that explains how apoE is recognized by the members of the LDL receptor family.

## Results

### Preparation of homogeneous apoE4 rHDL

**Fig 2:**
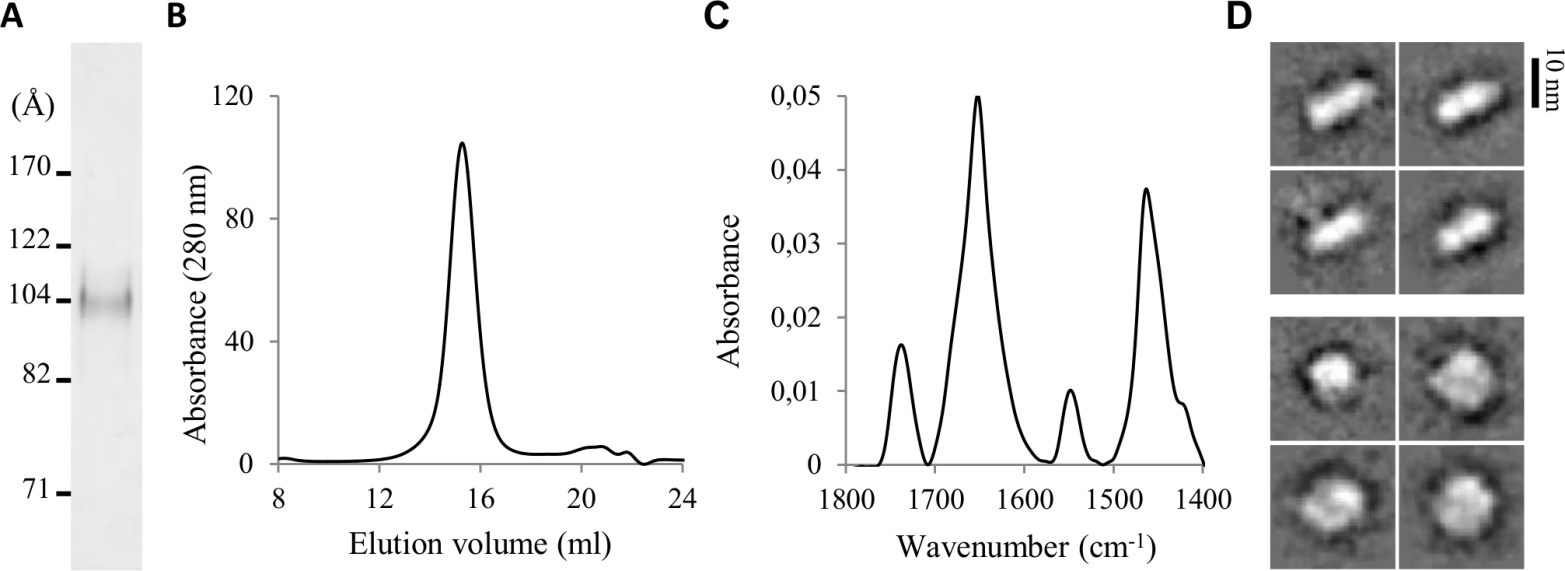
Characterization of apoE4/POPC rHDL. (A) Native PAGE gradient (3.5–13%) revealed with Coomassie blue staining. (B) Size-exclusion chromatogram after sodium cholate dialysis. (C) Infrared spectrum in the 1800–1400 cm^-1^ region. (D) Selected class-averages of cryo-TEM images evidencing side (upper) and top (lower) views of the nanodisc.

ApoE can bind to lipoproteins of variable sizes and shapes due to its conformational flexibility [42]. To obtain detailed information on lipidated apoE4 conformation, it was desirable to obtain highly homogeneous lipoproteins, in order to stabilize a uniform apoE4 conformation. For the preparation of such rHDL, we used the cholate dialysis method [43] and 1-palmitoyl-2-oleoyl-*sn*-glycero-3-phosphocholine (POPC) as a source of phospholipids. The initial lipid:protein molar ratio was optimized to enhance homogeneity of apoE4/POPC particles by following the resulting rHDL migration on native PAGE (S1 Fig). At an initial apoE4/POPC molar ratio of 1:110, a single population was apparent, displaying a Stokes diameter of ~105 Å (Fig 2A). A single population was also detected by gel filtration of apoE4/POPC rHDL (Fig 2B). Finally, quantification of the protein content indicated that the apoE4/POPC reconstitution allowed recovering up to ~50% of the protein initially engaged in rHDL. These reconstitution parameters allowed us to prepare highly homogeneous apoE4/POPC rHDL particles in a reproducible manner. To provide detailed input data for the structural modeling of lipid-bound apoE4, we extensively characterized their composition and shape.

Quantification of the concentration of lipid by phosphorus assay revealed that each particle contained about 200 POPC molecules. Chemical XL with large excess of bis(sulfosuccinimidyl) suberate (BS^3^; molar ratio BS^3^:apoE4 200:1) followed by SDS-PAGE analysis resulted in a single apoE4 dimer band at approximately 70 kDa, indicating that two molecules of apoE4 were present on each apoE4/POPC rHDL. Infrared measurement revealed a sharp peak for the amide I band (1700–1600 cm^-1^) centered at 1652 cm^-1^ characteristic of α-helical structures [44] (Fig 2C). An α–helical content of approximately 60% was estimated by curve-fitting of the amide I band. ApoE4/POPC rHDL were next characterized by both negative staining (NS) and cryo-transmission electron microscopy (TEM). Most representative class-averages of NS-TEM images revealed that apoE4/POPC rHDL mainly appeared to be of circular shape with a diameter of 115 ± 10 Å (S2A Fig). To determine the overall shape of the rHDL particles in hydrated state and to avoid possible artifacts due to sample drying and heavy metals on the observed shape, we visualized the apoE4/POPC rHDL by cryo-TEM (S2B Fig). The homogeneity of the apoE4/POPC reconstitution enabled us to perform single particle analysis. Two-dimensional averages identified both top and side views of the rHDL particles (Fig 2D). These projections displayed a diameter similar to the one previously measured in NS-TEM and a thickness of 50 ± 10 Å, in good agreement with the expected thickness of a POPC bilayer [45]. NS- and cryo-TEM images therefore strongly support a discoidal shape for the apoE4/POPC rHDL. These particles will further be designated as apoE4 nanodiscs in this work.

### XL-MS study of apoE4 nanodiscs

**Fig 3:**
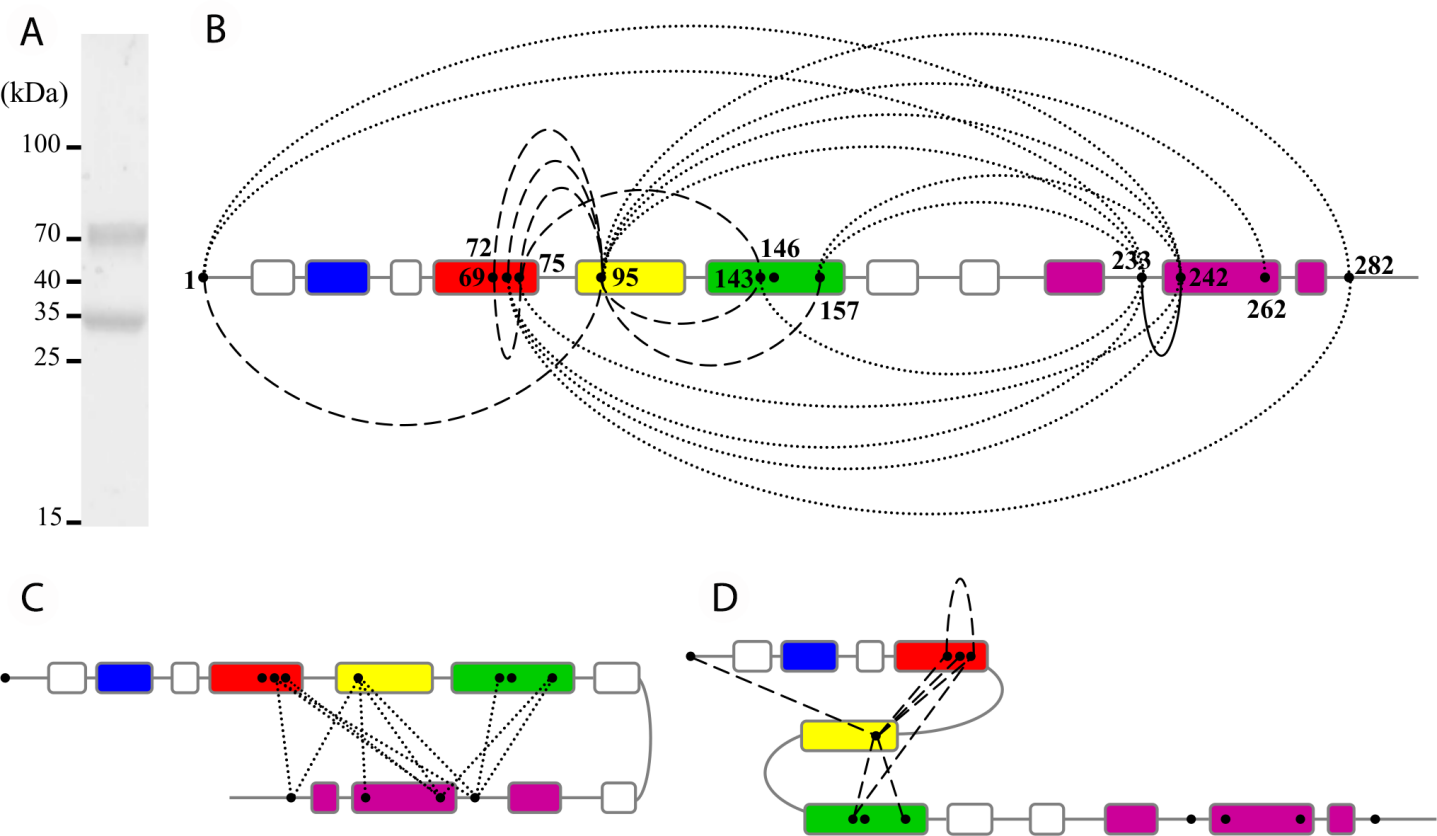
XL-MS of apoE4 nanodiscs. (A) ApoE4 nanodiscs cross-linked by DSS and analyzed by SDS-PAGE (8%) stained with Coomassie blue. Cross-linking was carried out with a 8:1 molar ratio of DSS:apoE4 at 0.5 mg/ml apoE4 concentration. (B) Overview of the intramolecular XLs obtained for apoE4 nanodiscs after XL-MS analysis of the apoE4 monomeric band. Helices 1, 2, 3 and 4 from the NT domain are colored in blue, red, yellow and green respectively. The CT domain helices are colored in purple. Intra-NT domain XLs are shown by dashed lines, intra-CT domain XL by a straight line and NT to CT domain XLs by dotted lines. (C) Model of a hairpin conformation suggested by the extensive number and variety of NT to CT domain XLs. (D) Compact state of the NT domain suggested by the different intra-NT domain XLs.

The apoE4 protein conformation at the surface of nanodiscs was investigated by XL-MS using the homobifunctional disuccinimidyl suberate (DSS) cross-linker that reacts with primary amino groups (Lys residues and protein N-termini). The extended Cα-Cα distance for lysine pairs that can be cross-linked by DSS is usually considered to have an upper limit of about 30 Å [46,47]. An equimolar mixture of light DSS (DSS-H12) and heavy DSS (DSS-D12) was used, providing a unique isotopic signature to cross-linked peptides and facilitating their detection and identification by MS [48,49]. The apoE4 molecules at the surface of the nanodiscs were cross-linked with 8 moles of DSS for 1 mole of apoE4. The low DSS/apoE4 molar ratio was chosen to minimize the risk of disturbing the structure adopted by apoE4 in the nanodiscs. The resulting species were isolated by SDS-PAGE revealing two bands of comparable intensity at approximatively 35 and 70 kDa, which were assigned to cross-linked monomeric apoE4 and cross-linked dimeric apoE4, respectively (Fig 3A). To generate exclusively unambiguous intramolecular XL products, the monomeric apoE4 band at 35 kDa was processed by in-gel digestion with trypsin and analyzed by liquid chromatography MS/MS. The resulting fragment ion spectra were analyzed using the dedicated software pipeline *xQuest*/*xProphet* [46,48]. 27 cross-linked peptides were identified for monomeric apoE4 (S1 Table), which corresponded to 22 unique Lys-Lys distance restraints (Fig 3B and Table 1).

**Table 1:** Lys-Lys residues cross-linked in apoE4 nanodiscs and validation of the opened hairpin and compact hairpin models by XL distance compatibility^a^.

Cross-linked residues	Opened hairpin model	Compact hairpin model
Lys1-Lys95	Yes	Yes
Lys1-Lys233	No	Yes
Lys1-Lys242	No	Yes
Lys69-Lys75	Yes	Yes
Lys69-Lys95	Yes	Yes
Lys72-Lys95	Yes	Yes
Lys72-Lys233	No	Yes
Lys72-Lys242	No	Yes
Lys72-Lys282	Yes	No
Lys75-Lys95	Yes	Yes
Lys75-Lys143	No	Yes
Lys75-Lys242	No	Yes
Lys95-Lys143	No	Yes
Lys95-Lys157	No	Yes
Lys95-Lys233	No	Yes
Lys95-Lys242	No	Yes
Lys95-Lys262	Yes	No
Lys95-Lys282	Yes	No
Lys143-Lys233	Yes	Yes
Lys157-Lys233	Yes	Yes
Lys157-Lys242	Yes	Yes
Lys233-Lys242	Yes	Yes
Total	12/22	19/22

^a^For DSS reagent, it is generally assumed that the Euclidean distance between a pair of cross-linked Lys Cα atoms is lower than 30 Å [46, 47].

Evaluation of the intramolecular XL data set obtained for the apoE4 nanodiscs revealed that both CT and NT regions of the protein are covered by the ensemble of XLs, with 11 out of the 12 apoE Lys residues involved in at least one XL. The XLs can be classified into two main categories. The first, and largest, group comprises XLs that were formed between Lys residues located in the NT domain and the CT domain (Fig 3B and 3C, dotted lines). The second group contains pairs of Lys residues belonging to the NT domain only, the vast majority connecting different helices forming the four-helix bundle adopted by apoE in its soluble form (Fig 3B and 3D, dashed lines). Topological information on the conformation of lipidated apoE4 could be deduced from the distance restraints derived from the XL data. The distribution and number of intramolecular XLs between Lys residues of the NT and CT domains were inconsistent with a completely extended conformation of apoE4 at the surface of the nanodiscs. They rather suggested a hairpin conformation (Fig 3C). Besides, the scattering and number of intra-NT domain XLs were indicative of a relatively compact state of the NT helix bundle (Fig 3D).

Once the in-depth experimental characterization of the nanodiscs was achieved, we set out to generate a model of apoE4 bound to rHDL. To do so a two-step procedure was set up (Fig 1): first, monomeric conformations of apoE4 were constructed by molecular modeling using experimental data to guide the modeling process. Then, dimer assemblies of these monomer structures were wrapped around an explicit lipid disc and the evolution over time of these systems was investigated by molecular dynamics simulations.

### XL guided molecular modeling of monomeric apoE4 structures

In a first modeling approach, we directly used all the intramolecular XLs as long and medium-range distance restraints so as to generate a structural model of monomeric lipidated apoE4. However, this attempt was unsuccessful as the ensemble of XLs restraints could not generate any concluding structures that would fit the experimental characterization of the nanodiscs (shape and size). From this first approach, it appeared evident that the ensemble of XL data would not be satisfied by a single ultimate model, hinting at the presence of at least one alternative conformation.

We therefore devised a second approach in which here-acquired structural data were rationalized in the light of current knowledge on lipidated apoE to narrow the range of conformational states apoE4 could adopt at the surface of nanodiscs (Fig 1). They were implicitly included in sets of constraints for the structure generation (S1 Text and S2 Table). First, to fulfill the hairpin conformation suggested by the NT-CT spatial proximity, evidenced by our XL-MS data (Fig 3C), we inserted a hinge, allowing the CT domain to fold back along the NT domain. To determine the apex of the hairpin, we tested three different hinge positions in the non-structured portions of apoE4 connecting the NT and CT domains (res. 164 to 168, 186 to 193, or 201 to 208). Although our XL data pointed toward a relatively compact conformation of the NT domain (Fig 3D), we conjectured that an apoE4 NT domain conformation completely folded as in solution would be hardly compatible with a receptor active conformation, as it is commonly accepted that opening of the NT bundle upon lipid interaction is a prerequisite for exposure of NT helix 4 containing the region involved in recognition of LDL receptors [19]. Therefore, based on literature [18,22,28,29], we decided to partially open the NT four-helix bundle by unfurling the turn in between NT helices 3 and 4 and aligning these two helices with the CT domain in a hairpin conformation by using a zipping procedure. Second, we maintained NT helices 1 to 3 bundled together by applying a zipping procedure between NT helices 2 and 3, thus promoting their spatial proximity to comply with the XL-MS data and to place NT helix 2 outside of the implicit lipid disc. On the other hand, due to the lack of XL data for NT helix 1, which does not contain any Lys residue, preventing us to rule on its position, we chose to keep this helix in contact with NT helix 2 by using the distances and angles from the NMR study of full length mutated apoE3 [18]. A partially opened state comprising a NT three-helix bundle with NT helix 4 detached was hence generated. Finally, we imposed a curvature to adapt the conformation of apoE4 molecules to the experimental discoidal shape of the nanodiscs and applied a distance constraint to move the flexible CT end outside of the nanodisc.

**Fig 4:**
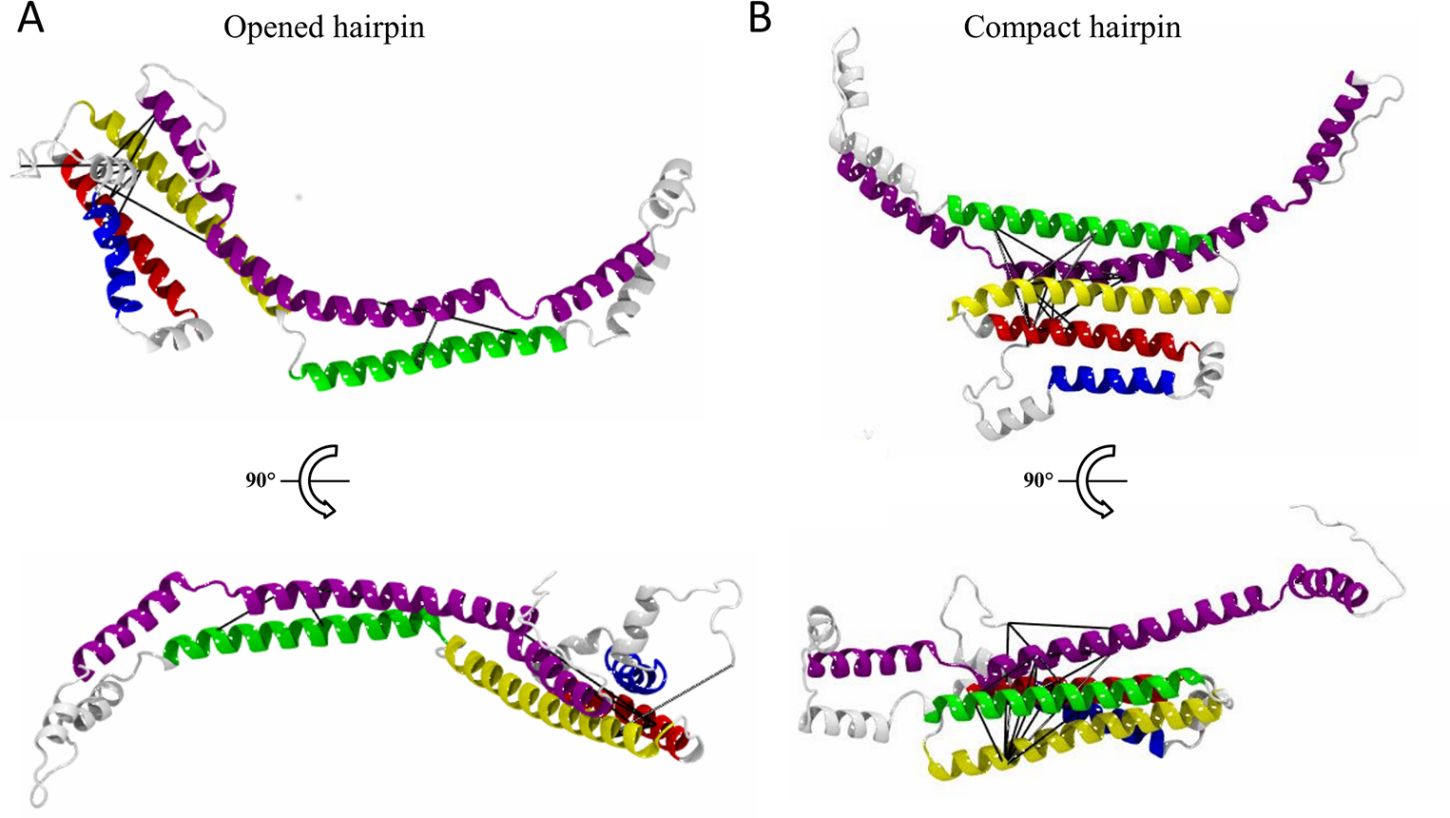
Structural models of apoE4 at the surface of a nanodisc. The structural modeling produces two different models of monomeric lipidated apoE4: the opened hairpin (A) and the compact hairpin (B) models that fulfill 12 and 19 out of 22 experimental XL restraints, respectively. For each model two views are shown, from the top of the lipid disc (upper) and rotated by 90° reflecting a view from the inside of the lipid bilayer of the nanodisc (lower). Cross-links with satisfied distance restraints within their respective models are shown as black lines. The secondary structure elements are colored as in Fig 3.

Validation of our models by the XL data revealed that, from the structures generated with the three different hinge positions, the model featuring a hairpin structure containing the hinge formed by res. 186 to 193 best matched the XL pattern, satisfying 12 out of 22 XLs (Table 1). This model was named “opened hairpin” model (Fig 4A) and the selected hairpin apex placed NT helices 3 and 4 in juxtaposition to the CT domain, in good agreement with 6 XLs (out of 11) formed between these two domains (Table 1). Nevertheless, the opened hairpin model left out 10 XLs that failed to comply with its structure. These non-satisfied XL were either intra NT domain (helices 2/3 connected to helix 4) or NT-CT domains XL (helices 2/3 connected to a different region of the CT domain) (Table 1). Careful inspection of the opened hairpin model suggested that these XLs were likely to be satisfied if the NT domain adopted a four-helix bundle. We thus constructed a second monomeric apoE4 model, using the same hinge region (res. 186 to 193) but adjusted the constraint list (S2 Table) to retain a compact state of the NT domain bundle. Remarkably, in this second model, named “compact hairpin” model in the following (Fig 4B), 19 out of the 22 identified XLs were validated (Table 1). The three non-satisfied XLs involved a subset of the NT-CT links (helices 2/3 with res. 262 and 282 of the CT domain) that otherwise supported the opened hairpin model. The two conformations proposed here may therefore represent distinct states of lipidated apoE4 that dynamically co-exist in solution.

### Construction of lipidated apoE4 nanodiscs and their dynamical behavior

Both the opened and compact hairpin monomeric models were dimerized in either a head-to-head or head-to-tail orientation. They were wrapped around a solvated POPC disc producing four different molecular systems (S1 Text and S3 Fig). In all 4 setups, the final number of lipids contained in the nanodisc is in good agreement with the experimental values we measured, providing a first validation of our models before we further studied their dynamic behavior using molecular dynamics simulation.

**Fig 5:**
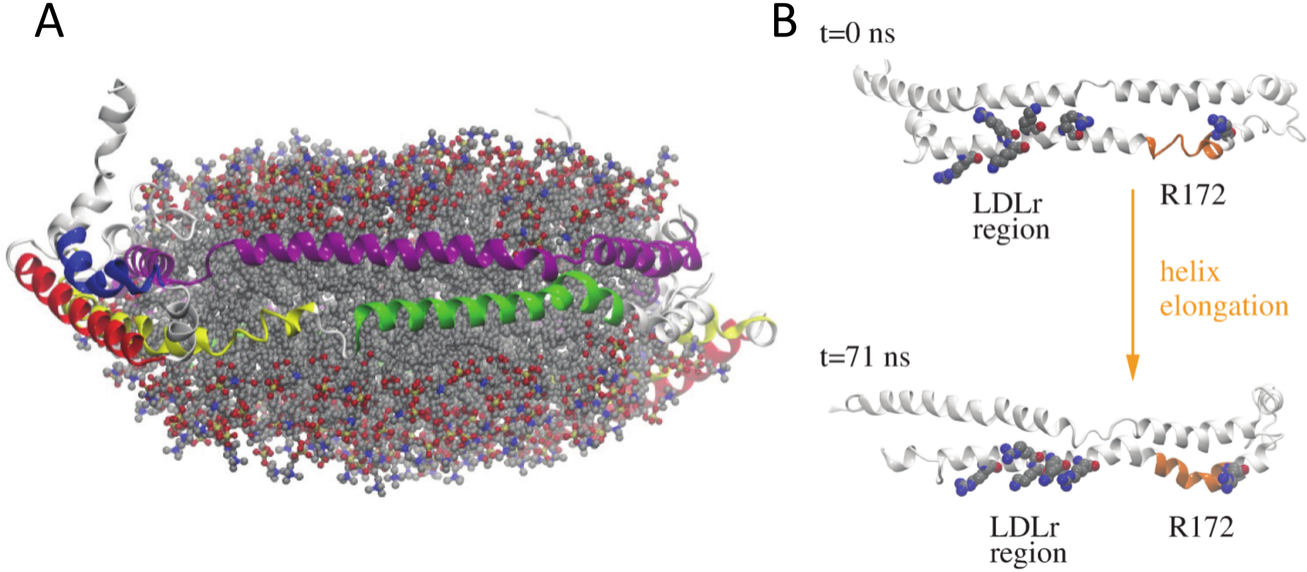
3D model of apoE4 nanodisc and extension of NT helix 4. (A) Representative conformation of one of the molecular dynamics simulations showing the head-to-tail opened hairpin model. The proteins are shown as cartoon and the secondary structure elements are colored as in Fig 3. The lipids are shown as semi-transparent ball-and-stick models colored by atom type (grey, carbon; blue, nitrogen; red, oxygen; yellow, phosphorus). (B) The α-helical content of the apoE4 region 120 to 270 in the initial structure and at the end of the molecular dynamics simulation shows the conversion of a short stretch (res. 164 to 168) at the end of NT helix 4 from a random coil to an α-helical conformation (highlighted in orange). In addition, the six residues, including R172, known to be important for binding to LDL receptors undergo a reorientation; they are highlighted as van der Waals spheres colored by atom type.

In the first nanoseconds of the trajectories, the amphipathic α-helices were observed to rearrange so as to more efficiently protect the hydrophobic acyl chains of the lipids located at the edge of the nanodiscs from the solvent. By adjusting their α-helical segments contacting the lipids, two apoE molecules are able to accommodate the number of lipids contained in each lipoprotein particle and match the average diameters of the nanodiscs as they were observed in this study by native PAGE (Fig 2A), NS- and cryo-TEM (Fig 2D and S2 Fig). Further, our 75-ns long trajectories highlighted that the lipid structures kept their disc shape in all cases (Fig 5A and S4 Fig) and the majority of the XLs remained satisfied at the end of our simulations (S3 Table). The α–helical content at the end of the simulations calculated with DSSP [50] ranged between 51% and 66% in good agreement with the 60% estimated from our infrared measurements. No significant differences could be evidenced between the systems featuring either a head-to-head or head-to-tail apoE dimer and we therefore could not discriminate between both orientations. However, during the trajectories local changes in the secondary structure were observed in some regions of the protein. Remarkably, a short stretch (res. 164 to 168) at the end of NT helix 4 switched from a random coil to an α-helical conformation and remained α-helical for the rest of the simulation in one of the monomers in all models (Fig 5B). This structural change, close to the binding region to LDL receptors, promoted an extension of helix 4 resulting in a long amphipathic helix spanning res. 131 to 180 (Fig 5B). Furthermore, upon this change Arg172, known to be involved in the recognition of LDL receptors [51] and other upstream basic residues, also known to interact with the receptor [52], underwent a reorientation leading to their respective alignment (Fig 5B). Comparison of the solvent accessibility of these residues in our two models (S5 Fig) indicated that, while most residues binding to the LDL receptors featured a low accessibility in the compact hairpin model, they really pointed into the solvent in the opened hairpin model regardless of the dimer arrangement. Therefore, although both conformations may co-exist in solution, they may exert variable binding activities towards receptor recognition with the opened hairpin model representing the active conformation of lipidated apoE4.

## Discussion

High resolution structural data are generally the key for a molecular understanding of the function of a protein and protein complex. However, the structure of certain protein assemblies, such as apolipoproteins associated with lipoprotein particles, are yet difficult or simply impossible to determine by traditional high resolution structural methods. Nowadays, an increasing body of literature advocates the integration of low resolution experimental data with bioinformatics tools for protein structure determination of challenging protein assemblies [53,54]. Among these hybrid methods, the integration of distance restraints provided by XL-MS data in structural modeling represents a very promising avenue as previously demonstrated [41,55–57], also for the structural organization of lipid-free and lipid-bound apolipoproteins [37,55,58,59]. As described in our study, in order to determine the structure of the active form of apoE4 associated with discoidal rHDL particles, we developed an approach where we used NS-, and cryo-TEM, XL-MS and other experimental techniques in combination with molecular modeling and simulation. From our integrated workflow (Fig 1), we modeled 3D structures of apoE4 associated to a nanodisc. Our resulting models differ from previously proposed conformations of lipidated apoE4 and allow us to propose novel mechanisms of regulation coupling accessibility and activation of the receptor binding abilities of lipidated apoE4.

### Organization of apoE4 molecules at the surface of nanodisc

The XL-MS distance restraints obtained here from the cross-linked monomeric apoE4 molecules argued against a model where apoE could adopt a completely extended structure surrounding the nanodisc, with two molecules of apoE running along each other in a ‘double-belt’ organization as was proposed previously [22]. A large subset of our intramolecular XL data rather inferred a hairpin fold of lipidated apoE as previously proposed in other studies [29,31]. Alike previous models of full-length apoE, our XLs implied the hinge of the hairpin to be situated in the unstructured region connecting the NT and CT domains but with subtle differences resulting in significant structural and mechanistic implications. Specifically, in the so far most detailed Xray/EPR model of lipidated apoE4 [30, 31], the hinge is situated at res. 162 to 169 (vs res. 186 to 193 here) and suggested to bring in close proximity regions that are known to be important for the interaction with LDL receptors, the region spanning res. 134 to 150 and Arg172. However, due to the hinge location in this model, the α-helical extension of NT helix 4, suggested to be essential for receptor binding activity [24,25], is no longer possible. This hinge location was also not supported here, as the model we built with the hinge on res. 164 to 168 only satisfied 9 out of the 22 identified XLs. In spite of the difference in hinge localization, spatial proximity of significant pairs of residues could be reconciled between our and previous hairpin models. For instance, for apoE4, the spatial proximity of two residues, Arg61 and Glu255, that are proposed to form a salt bridge promotion the interaction between NT and CT domains in the lipid-free form [60], was confirmed to be maintained in the lipid-bound state in discoidal particles [29]. The proximity of these residues was also preserved here, thanks to the partially closed conformation of the NT domain. Further, a significant number of EPR constraints [31] were also validated in our models, including the intramolecular spatial proximity of residues 76/77 with residues 239/241 that were established in our study to be intramolecular by the selection of the monomeric band for in-gel digestion (Fig 3A). The here-produced hairpin models thus allow at the same time both spatial proximity of recognized pairs of important residues in CT and NT regions and the opportunity for the extension of helix 4 needed for the recognition of LDL receptors (Fig 5B). However, a limitation of our study is that, in the current setting, we did not specifically discriminate between intra- and intermolecular cross-links within homodimeric apoE proteins and the respective organization of the two apoE molecules on the lipid particle could therefore not be deduced. The head-to-head and head-to-tail dimerizations, as presented in S3 Fig, therefore remain to a certain degree speculative.

### Two levels of accessibility of the LDL receptor binding region

**Fig 6:**
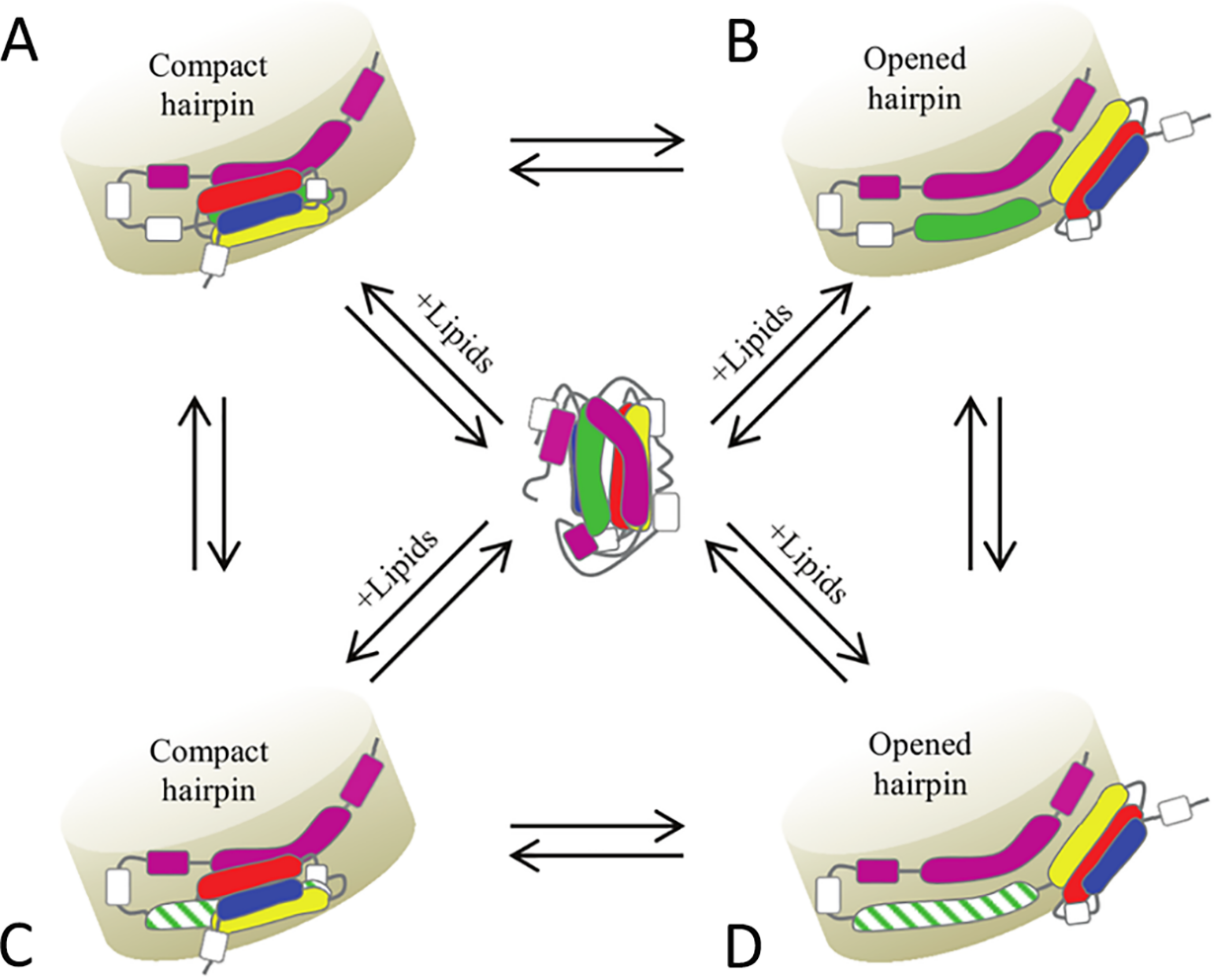
Mechanism of regulation and activation of lipidated apoE4. After encounter of lipids, two free apoE4 molecules will adopt either a compact hairpin (A) or opened hairpin (B) configuration around the nanodisc which is followed/concomitant with an elongation of NT helix 4 (C and D). Conversions of the two configurations are possible. On the opened hairpin configuration, the elongated NT helix 4 is accessible and recognized by the LDL receptors (D). The helices are colored as in Fig 3. The elongated NT helix 4 is highlighted in dashed green.

Two alternative models, featuring three or four bundled amphipathic helices from the NT domain, were constructed that together satisfied the ensemble of XL derived spatial restraints (Fig 4 and Table 1). The compact hairpin model features a NT four helix bundle laid along the CT domain and interacting with the lipids only *via* helix 4. In this conformation, helix 4, that contains essential residues for recognition of the members of the LDL receptor family [52], was shielded from the solvent by helix 3 (Fig 4B and S5 Fig). In the opened hairpin model, the turn between helices 3 and 4 was unfurled, in agreement with previous studies [18,28], and allowed an opening of the bundle with NT helix 3 now interacting with the lipids. This partial opening of the bundle was sufficient to expose helix 4 to the solvent (Fig 4A and S5 Fig). The opening movement from the compact to the opened hairpin model therefore provides us with a possible regulatory mechanism of apoE4 lipoproteins (Fig 6A and 6B). In contrast to earlier studies that indicated that the interaction of the NT domain with the lipids would engage an open and active conformation of the receptor binding region [18,26], our models strongly suggest that, in both the open and compact state, the NT domain of apoE is associated with lipids at the surface of the nanodisc. The domains outside the lipid disc in the compact hairpin model (S3C and S3D Fig) are not clearly resolved by NS-TEM (S2 Fig). Heterogeneity in the disc size, dynamic structure of NT domain switching between compact and open conformation, and small size of the folded domain preclude its visualization by single particle technique based on averaging of projections of individual aligned particles. Moreover NT helix 1 that was considered in our modeling as part of the NT helix bundle despite the absence of structural data could instead adopt a more extended conformation. Each of these two cases would then contribute to decrease the compactness of the NT portion that may be observed by NS-TEM.

We speculate that both the open and compact hairpin model co-exist in a dynamic equilibrium where the different forms could concurrently be captured by our XL experiments. Further, we propose that in presence of the receptor this equilibrium is shifted to the opened hairpin model, the model that represents the state accessible to LDL receptors, and therefore allows us to draw a mechanism of accessibility of the LDL receptor binding region (Fig 6A and 6B).

### Activation of LDL receptor binding abilities

A relatively small structural change, observed in all models during the molecular dynamics trajectories, elongated helix 4 and connected it with a subsequent small helix spanning res. 169 to 180, leading to the formation of a 50 residue-long amphipathic helix (res. 131 to 180) (Fig 5B). This helix extension upon lipidation has already been proposed experimentally by NMR and EPR [24,25] and it was suggested to act as a molecular switch that stably anchors the receptor binding region on the lipid surface or/and correctly positions residue 172 with other basic residues (in the region 136 to 150) known to be required for an optimal interaction with the LDL receptors [51,52]. These receptors share highly conserved structural domains, including ligand-binding domains containing cysteine-rich ligand binding type-A (LA) repeats. For the LDLr, the most prevalent member of this family of receptors, it is now well established that among the 7 LA repeats, LA5 is essential for binding of apoE lipoproteins [61] and that the pair LA4-LA5 is sufficient to bind apoE in rHDL [62]. The residues known to interact with the LDLr on apoE [51,52] span a too large region to be recognized by a single LA repeat of the LDLr and would thus allow for the binding of the two LA repeats to the same apoE molecule as we proposed earlier based on the lipid-bound structure of an apoE-derived peptide [25]. In addition, our study showed that the elongation of NT helix 4 upon lipidation led to a reorganization of the LDLr binding residues that could promote their binding to the LDLr LA4-LA5 repeats (Fig 5B). To support this hypothesis, we performed docking assays in which LA4 and LA5 were docked individually to such an elongated NT helix 4 (S4 Table). The results confirmed that the distance between the two docked modules was in agreement with the long loop between LA4 and LA5 repeats (S6 Fig). This distance is unique between this pair of LA repeats [63], highlighting its importance in lipidated apoE recognition. Contrary to the soluble form of apoE4, the elongation of NT helix 4 conferred upon lipidation would therefore represent an additional prerequisite for binding to LDL receptors (Fig 6D).

In summary, our data advocate that several requirements need to be met to provide a fully receptor-active apoE (Fig 6D): lipid binding, exposure of the receptor binding region and elongation of NT helix 4. We speculate that the here proposed compact hairpin model is a stable conformation co-existing with the active, receptor-competent open structure, explaining why these two alternative conformations could be trapped in the XL-MS experiments. Both conformations may therefore be part of a regulation mechanism of apoE function at the surface of lipids. Our work represents a building stone towards a better understanding of the strong anti-atherogenic effect of apoE and the models we are proposing could prove useful in the study of lipidated apoE in totally different contexts, such as understanding its role in Alzheimer’s disease. Overall our hybrid approach, compatible with the presence of lipids, results in 3D structures of lipidated apoE4 that represents the most comprehensive model of the active form of apoE4 to date and might be applied to the study of other (membrane) proteins where such complementary low resolution structural data are available.

## Materials and methods

### Chemicals and reagents

Unless otherwise stated, all chemicals were obtained from Sigma-Aldrich at the highest purity available. Water was double-distilled and deionized using a Milli-Q system (Millipore).

### Expression and purification of apoE4

The human full-length apoE4 gene fused to a self-cleavable intein tag and a chitin binding domain cloned into a pTYB2 vector was a kind gift of Dr. Vasanthy Narayanaswami (University of Long Beach, California, U.S.A.). ApoE4 was expressed in a T7 expression strain of *Escherichia coli* (ER2566) in 2xYT medium by the addition of isopropyl β-D-thiogalactopyranoside. Pelleted cells were resuspended in buffer A (20 mM HEPES, 500 mM NaCl, 1 mM EDTA, pH 8), supplemented with 0.5% (v/v) triton-X-100 (TX100) and anti-proteases (cOmplete EDTA-free protease inhibitor cocktail, Roche), and apoE4 was released by high-pressure homogenizer. The protein was purified following standardized protocols previously described for intein-labelled proteins [64]. Briefly, the clarified cell lysate was loaded onto chitin beads (Impact system, New England Biolabs) equilibrated with 5 column volumes (CV) of buffer A containing 0.5% (v/v) TX100 and incubated at 4°C during 1 h on a rotating wheel. The flow through was discarded and the beads were washed with 10 CV of buffer A containing 0.3% (v/v) TX100. ApoE4 was released by incubation of the chitin beads with buffer A containing 30 mM dithiothreitol (DTT) at 4°C during 40 h and finally eluted with 3 CV of buffer A containing 5 mM DTT. ApoE4 was then buffer exchanged against buffer B (20 mM ammonium bicarbonate, pH 8) with PD-10 desalting column (GE healthcare) and lyophilized overnight. Prior to utilization, lyophilized apoE4 was solubilized in buffer C (20 mM HEPES, 150 mM NaCl, pH 7.4) containing 6 M guanidine-HCl and further purified by size exclusion chromatography on a Superose 6 matrix (GE Healthcare) eluted with buffer C containing 4 M guanidine-HCl. Fractions containing apoE4 were pooled together and dialyzed against buffer B during 48 h at 4°C. ApoE4 concentration and purity were assessed by absorbance at 280 nm and SDS-PAGE.

### Preparation of apoE4 rHDL

ApoE4 rHDL were formed using 1-palmitoyl-2-oleoyl-*sn*-glycero-3-phosphocholine (POPC, Avanti polar lipids) following a modified version of the protocol initially developed for apoA-I by Matz and Jonas [43]. POPC solubilized in chloroform was dried under nitrogen and resuspended to a concentration of 20 mg/ml in buffer C. Sodium cholate was added at a POPC:sodium cholate molar ratio of 1:2 and the mixture was sonicated for 1 h with vortexing every 15 min. ApoE4 was added to the mixture at different molar ratio and incubated overnight at 4°C on a rotating wheel. Sodium cholate was eliminated by dialysis during 24 h against buffer B (3 buffer exchange). Samples were purified on a Superose 6 matrix eluted with buffer C. Fractions containing apoE4 rHDL were pooled together and concentrated by filtration up to an apoE4 concentration of 0.5 mg/ml (Vivaspin 6, 50 K MWCO, Sartorius). The homogeneity and the size distribution of the rHDL were both assessed by blue native PAGE (S1 Fig) while their apoE4 and POPC content was evaluated by measuring the concentration of proteins and lipids by absorbance at 280 nm and phosphorus assay [65], respectively.

### Electrophoresis

Blue native PAGE (3.5–13%) electrophoresis was realized following a procedure described in [66] using HMW Native Marker Kit (GE Healthcare) for protein standards. SDS-PAGE (8%) was realized according to [67] using prestained protein ladder (Fermentas) as molecular weight size marker. Both blue native PAGE and SDS-PAGE were revealed using Coomassie blue staining.

### Infrared spectroscopy

Infrared spectroscopy was performed in attenuated total reflection mode and infrared spectrum was recorded on an Equinox 55 spectrophotometer (Bruker Optics). Measurement was made at room temperature by spreading 2 μL of apoE4/POPC rHDL solution (0.5 mg/ml) on the surface of the internal reflection element made of a diamond crystal. Excess water was removed under nitrogen flow. The spectrum represents the mean of 256 spectra recorded at a 2 cm^-1^ resolution. Data were analyzed using Kinetics software (SFMB, Brussels, Belgium) and processed for baseline correction and subtraction of the water vapor contribution. Curve-fitting on the non-deconvoluted spectrum was performed to determine the global secondary structure content of a protein. The proportion of a particular structure is computed to be the sum of the area of all the fitted bands having their maximum in the frequency region where that structure occurs divided by the total area of the amide I band between 1700 and 1600 cm^-1^. They were chosen by the program on the basis of the shape of the most deconvoluted spectrum (α-helices and random coil absorb at 1637–1662 cm^-1^, turns at 1662–1682 cm^-1^ and β-sheets at 1613–1637 cm^-1^ and 1682–1689 cm^-1^).

### Transmission electron microscopy

TEM data were collected at a nominal magnification of 60,000 and pixel size 1.9 Å/pix on a JEM-1400 (JEOL) operating at 120 kV with a LaB_6_ filament and equipped with a CMOS TemCam-416 4016x4016 camera (TVIPS). For NS-TEM, grids were glow discharged system and 2 μl of sample at concentration of 0.01 mg/ml was applied and stained with uranyl formate. Images were collected at defocus between 1 and 2 μm. A total of 6,766 particles were picked manually. The set of particles was first classified with multiple cycles of k-means classification and multi-reference alignment using SPARX with the exclusion of particles less representative. In complement, a second set of classifications was performed using EMAN2 [68] resulting in 25 class-averages corresponding to 1188 symmetric top-view particles. For cryo-TEM, a frozen-hydrated grid was prepared by blotting 2 μl of sample (1 mg/ml) on a Quantifoil holey carbon-film-coated 400-mesh copper grids and plunge-frozen. From a total of 660 images, with an average dose of 41 electrons/Å^2^, 29,134 particles were carefully manually selected. The particles were classified using EMAN2. The final representative class-averages were calculated from 10,249 particles.

### Chemical cross-linking coupled with mass spectrometry

XL-MS analysis was carried out essentially as described elsewhere [49]. Briefly, an 8-fold molar excess of DSS (Creative Molecule Inc.) over apoE4 concentration was added to the apoE4 nanodiscs. The mixture was incubated for 30 min at 37°C and the XL reaction was quenched by the addition of ammonium bicarbonate to a final concentration of 50 mM for 10 min at room temperature. The products resulting from the XL reaction were separated by SDS-PAGE and visualized with Coomassie blue staining. Bands containing the cross-linked species of interest were sliced from the gel into cubes of 1 mm^3^, transferred into protein low binding tubes (Eppendorf) and submitted to in-gel digestion [69] using trypsin (Promega). MS analysis was carried out on a Thermo Orbitrap Elite mass spectrometer (Thermo Scientific) and data analysis was performed using *xQuest* [48]. False discovery rates (FDR) and delta score (*delta*S) of cross-linked peptides were assigned using *xProphet* [46]. Cross-linked peptides that were identified with an assigned FDR below 5% and a *delta*S below 95% were selected for this study (S1 Table). In all cases the FDR, which denotes the false-discovery rate as calculated by *xProphet* [46], was equal to zero. All selected XLs were further analyzed by visual inspection in order to ensure good matches of ion series on both cross-linked peptide chains for the most abundant peaks. Lys146 was not detected as a XL site. This most likely resulted from trypsin digestion producing a dipeptide which is too short to be considered by our applied XL-MS method in order to ensure good matches of cross-linked peptides.

### Molecular modeling of the monomeric lipidated apoE4

Based on intramolecular XLs and information on the shape/organization of the apoE4 nanodiscs, the structure of an apoE4 monomer surrounding an implicit POPC disc was modeled with CNSsolve [70,71]. The modeling procedure is described in Supporting Information S1 Text.

### Molecular dynamics simulations of lipidated apoE4 dimers

All molecular dynamics calculations were performed in the isothermal-isobaric ensembles at 300 K with the program NAMD2.9 [72]. The CHARMM 27 force-field [73,74] with CMAP corrections [75] was used for protein, water and ions and a united atom force field [76] described the lipid molecules. The protocols of the molecular dynamics simulations and of the molecular dynamics trajectory analysis are described in the Supporting Information S1 Text.

### LA4/LA5 repeats docking details

LDLr LA repeats LA4 (res. 179–214) and LA5 (res. 121–167), as extracted from the first and representative conformation in the NMR structure (PDB code 2LGP) [77], were docked individually to a part of apoE4 (res. 125–185) containing the elongated helix 4 extracted from one molecule of apoE4 in the head-to-head opened hairpin model using the Haddock web server [78]. LA4/LA5 repeats were docked individually (and not together) as the loop between the two is highly flexible. In accordance to an earlier docking study [79] the LA5 module was docked to apoE4 using four unambiguous constraints (S4 Table). For the docking of the LA4 module to apoE4 ambiguous constraints were used in accordance to mutational data [51] and structural data of LA4 repeat bound to other proteins [80–82] (S4 Table). For the individual dockings of LA5/apoE4 and LA4/apoE4, 4 and 40 poses were obtained respectively. Each possible combination of LA4/LA5 repeats pairing was screened for the distance between the backbone carbon atom of residues Y167 (LA4) and F179 (LA5). The shortest distance between these residues is about 35 Å (S6 Fig), a value in good agreement with the loop length between LA4 and LA5 repeats (12 residues).

## Supporting information

S1 Text Molecular modeling of the monomeric lipidated apoE4 and molecular dynamics simulations of lipidated apoE4 dimers and trajectory analysis. (DOCX)

S1 Fig Reconstitution of apoE4/POPC particles. Samples were run on a native PAGE gradient (3.5–13%) and revealed with a Coomassie blue staining. Lanes 1 to 9: apoE4 incubated with 80-, 90-, 100-, 110-, 120-, 130-, 140-, 150-, and 160-fold molar excess of POPC.
(TIFF)

S2 Fig Identification of the shape of apoE4/POPC rHDL. Negative-stain TEM (A) and cryo-TEM (B) images (left) and selected class-averages (right) of the reconstituted apoE4/POPC particles.
(TIFF)

S3 Fig Representations of the apoE4 nanodisc configurations. The top (left) and side (right) views of the head-to-tail (A) and head-to-head (B) opened hairpin systems and the head-to-tail (C) and head-to-head (D) compact hairpin systems are depicted. The POPC lipids are shown as van der Waals spheres and the protein as cartoon colored as in Fig 3 have been used. In addition, the protein surface is shown in transparent.
(TIFF)

S4 Fig The averaged density profile of the lipids and protein extracted from molecular dynamics. The average density function of the lipid head (green) and tail groups (red), and the protein (blue) is plotted against the principal axis (z) of the system. The average density profile computed for the lipids and the protein showed a stable co-localization of both proteins and lipid acyl chains. These data were extracted from the 75-ns long molecular dynamics trajectory of the head-to-tail opened hairpin system. The curves are representative for all four simulations.
(TIFF)

S5 Fig The LDL receptor binding region is accessible in the opened hairpin model. Normalized SASA values of positively charged residues involved in recognition of LDL receptors. Accessibility values are averaged over the two apoE4 molecules present at the surface of the discs. For each configuration (H-to-H, head-to-head; H-to-T, head-to-tail) the SASA was averaged over the 75 ns long molecular dynamics simulations. SASA values were also calculated for the full-length mutated apoE3 structure (PDB code 2L7B) and the apoE3 NT domain (PDB code 2KC3).
(TIFF)

S6 Fig The receptor modules LA4 and LA5 docked on the elongated helix 4 of apoE4. The docked pose of LA4 (purple surface/cartoon) and LA5 repeats (orange surface/cartoon) (PDB code 2LGP) leading to the shortest distance between the two modules is shown. On the apoE4 helix (white cartoon, res. 125–180), residues important for LDLr binding are highlighted in cyan. In addition, the calcium ions complexed by LA4 and LA5 repeats are depicted as green van der Waals spheres.
(TIFF)

S1 Table Intramolecular apoE4 XLs identified by XL-MS of cross-linked apoE4 nanodiscs. ^a^Absolute position of the cross-linked Lys residues and their localization in the secondary structure elements of apoE4 (NTD: N-terminal domain, CTD: C-terminal domain) color-coded as in Fig 3. ^b^Exact amino acid sequence of the cross-linked peptides with the cross-linked Lys residues highlighted in red. ^c^*xQuest*S is a weighted sum of four subscores (xcorrc, xcorrx, match-odds and TIC) that is used to assess the quality of the composite MS^2^ spectrum as calculated by *xQuest*. ^d^*delta*S gives the delta score of the respective XL and is a measure for how close the best assigned hit was scored in regard to the second best as calculated by *xProphet*.
(PDF)

S2 Table Set of constraints used to build the two different monomeric models. ^a^The constraints were always applied to the Cα-Cα distance.^b^Cα-Cα distances extracted from the NMR structure of soluble apoE3. Distances between residues were measured using VMD and averaged on the different NMR structures available.
(PDF)

S3 Table XLs fulfilled at the end of each of the four different molecular dynamics simulations. ^a^ **Green check mark**: Satisfied XL, **Red cross**: Non-satisfied XL, **-/-**: XL not consistent with the specified hairpin model. ^b^XLs are considered to be satisfied if the Euclidean distance between the two Cα atoms is lower than 30 Å in the last 1 ns of the simulation. ^c^For each configuration (H-to-H, head-to-head; H-to-T, head-to-tail) the fulfillment is given for Monomer1 / Monomer2 (Mon1 / Mon2).
(PDF)

S4 Table Distance constraints for the docking of LA5 and LA4 repeats to apoE4. For each constraint, the atoms of apoE4 and LA5/LA4 repeats are indicated. In all cases, the pair of atoms constrained should form a salt bridge and thus, the distance range was set to 2–5 Å with an optimal distance of 3 Å for each constrained. Constraints between apoE4 and LA4/LA5 repeats were treated ambiguously/unambiguously.
(PDF)
