# Supplementary material for: Lipidated apolipoprotein E4 structure and its receptor binding mechanism determined by a combined cross-linking coupled to mass spectrometry and molecular dynamics approach
Source: PLoS Comput Biol. 2018 Jun 22;14(6):e1006165. doi: 10.1371/journal.pcbi.1006165 (PMC6033463; doi:10.1371/journal.pcbi.1006165)
Supplement: S1 Text — (DOCX) [file pcbi.1006165.s001.docx]

**S1 Text.**

Molecular modeling of the monomeric lipidated apoE4.

First an extended conformation of apoE4 was generated by molecular modeling based on its sequence. Second, the helical stretches of apoE4 (residues 12 to 22, 25 to 42, 44 to 52, 54 to 80, 86 to 124, 129 to 163, 168 to 180, 191 to 198, 203 to 221, 226 to 264 and 267 to 285) were identified using the NMR structure of full length (mutant) apoE3 (PDB code: 2L7B) [18] in combination with the consensus position of helices in several other human apoE Xray structures (PDB codes: 2KC3, 1LPE, 1NFN, 1BZ4, 1OR2, 1OR3, 1LE4, 1B68, 1GS9, 1LE2, 1NFO, 1OEF and 1OEG; [15, 83-88]) and secondary structure predictions. Ten structures were calculated from the initial conformation using typical α-helical NOE constraints to generate these helical segments in a simulated annealing protocol: a high temperature annealing stage up to 1000 K (10000 steps each 5 fs) followed by a short slow-cool annealing stage down to 0 K (10000 steps each 5 fs). From these structures, the one featuring the best fit to the pre-defined constraints and the lowest internal energy (according to CNS internal energy function) was selected for the following molecular modeling step.

In a first approach, we used all the XLs identified within monomeric apoE4 as low-resolution distance restraints to generate a three-dimensional modeled structure of the lipidated apoE4 monomer. The restraints were defined as Euclidean distances between the Cα atoms of the cross-linked Lys residues with an upper bound value of 30 Å. This value corresponds to the sum of the length of the DSS spacer of about 11.4 Å and two times the length of a lysine side chain (6.5 Å) plus a correction accounting for the flexibility of the protein backbone (~6 Å) [89]. This first approach however failed to produce any concluding structures.

We then adopted a second approach in which the XLs were not used as input distance restraints anymore but were implicitly exploited to narrow the range of conformational states of lipidated apoE4. Previously known structural data on apoE and lipoprotein complexes were also included to assist in building the structure. Eventually, the XLs served for the validation of the generated models; a XL was satisfied if the Euclidean distance between the Cα atoms of the cross-linked Lys residues was below the upper bound value of 30 Å.

To calculate the initial apoE4 lipid-bound structures, two series of distance constraints were defined as followed (S2 Table):

Opened hairpin model

1°/ For the generation of a hairpin structure, we tested three different hinge positions in the region connecting the N-terminal (NT) with the C-terminal (CT) domains in unstructured stretches (hinge at res. 164–168, 186–193, or 201–208). As a consequence, NT and CT regions were paired together by zipping helix faces together (S2 Table - constraint set A, showing only the distance constraints used for the hinge formed by res. 186–193). This alignment was achieved by sequentially selecting residues that should face each other in the hairpin region and assigning a distance of 6 Å to the pair of Cα atoms of these residues. This zipping procedure produced the juxtaposition of NT helices 3 and 4 regions with the CT domain allowing the aligned regions to cover the lipid bilayer thickness and a concomitant orientation of the hydrophobic faces of the concerned apoE helices towards the lipids.

2°/ The NT helices 1–3 were kept bundled together and outside of the lipid disc. Because NT helix 3 interacts with the CT domain (see 1°/) and implicitly with the lipids, it prevented us to use NMR constraints to engage it with NT helices 1 and 2. Therefore, (*i*) we applied a zipping procedure, as used to introduce the hinge (described above in 1°/), by imposing distance constraints to pair helices 2 and 3 together (S2 Table - constraint set B). Carefully choosing the faces of the helices to be paired allowed us to ensure that helices 1 and 2 were left outside of the implicit lipid disc. Then (*ii*) a selection of distance constraints extracted from the NMR mutant apoE3 structure between NT helices 1 and 2 was defined to keep them bundled (S2 Table - constraint set C).

3°/ According to our native electrophoresis measurements of the apoE4 nanodiscs, the diameter of the particle was 105 ± 5 Å (Fig 2A). Therefore distance constraints were applied to bend the protein so that it matched the measured diameter of the nanodisc (S2 Table - constraint set D).

4°/ Finally, to keep the flexible CT end outside of the implicit lipid disc, we applied a distance constraint to pair it with the nearby NT domain region (S2 Table - constraint set E).

The hinge located at position 186–193 produced the model that best satisfied the XL list (12 out of the 22 experimental intramolecular XLs, Table 1) and featured an overall shape fitting a discoidal lipid bilayer.

Compact hairpin model

1°/ According to our results from the opened hairpin model, we placed the hinge on res. 186–193 by zipping the required NT and CT parts together (S2 Table - constraint set F), as described above, except that only NT helix 4 forms the hairpin structure together with the CT domain.

2°/ The NT domain was folded as a four-helix compact state outside of the lipid disc. Following the hairpin formation (1°/), NT helix 4 interacts with the CT domain and implicitly with the lipids which prevented us to use the NMR constraints to keep it bundled with NT helices 1-3. Consequently, (*i*) we applied distance constraints in order to pair NT helices 3 and 4 together by using a zipping procedure (S2 Table - constraint set G). Careful choice of the face of the helices to be paired, NT helix 3 with NT helix 4 and NT helix 2 with the CT domain (S2 Table - constraint set G), produces a conformational model maintaining helices 1 to 3 outside of the lipid disc. Then (*ii*) a selection of distance constraints extracted from the NMR mutant apoE3 structure for NT helices 1 to 3 were introduced to keep them bundled (S2 Table - constraint set H).

3°/ Identical constraints as the ones used for the opened hairpin model were introduced to impose a curvature to apoE4 matching the size of the lipid nanodisc (S2 Table - constraint set I).

4°/ Finally, to keep the flexible CT end outside of the implicit lipid disc, a distance constraint was applied to pair it with another nearby region of the CT domain (S2 Table - constraint set J).

Using the two sets of defined constraints (S2 Table), a simulated annealing procedure was carried out as described above, except that 40000 steps were used for the high temperature and slow-cool annealing stage, and 20 structures were generated. The structures that best fit the input data and fulfil the most XLs were refined using the respectively satisfied intramolecular XLs (Table 1), in addition to the original distance constraints (S2 Table) in a final annealing cycle. XL distance restraints were fixed at 30 Å (+ 0 Å, -24 Å) between the Cα of connected Lys residues. 50 structures were generated. Among the structures that best fit the experimental input data, the one with the lowest internal energy was considered further.

# Molecular dynamics simulations of lipidated apoE4 dimers and trajectory analysis.

**Molecular dynamics simulations of lipidated apoE4 dimers.** The opened hairpin and compact hairpin apoE4 monomeric models were each dimerized in a head-to-head or head-to-tail conformation using VMD [90]. The so generated four setups (head-to-head opened hairpin, head-to-tail opened hairpin, head-to-head compact hairpin and head-to-tail compact hairpin) were lipidated using a large hexagonal POPC bilayer produced using the membrane builder plug-in of the CHARMM-GUI webserver (<http://charmm-gui.org>) [91]. The lipid acyl chains were aligned perpendicularly (using VMD) with the α-helices of the protein dimers. Lipids overlaying protein residues or beyond the protein belt were manually removed. According to pK_a_ calculations performed using the program propKa 2.0 [92] on both monomeric apoE models, all ionizable residues were kept in their standard pH 7 protonation states. The protein lipid system was solvated, neutralized and a NaCl concentration of 0.15 M was added. Additional lipid molecules showing sterical hindrance with protein atoms after short molecular dynamics were removed. Following this step, each nanodisc contained approximately 200 POPC (200, 200, 199, and 200 for the head-to-head opened hairpin, head-to-tail opened hairpin, head-to-head compact hairpin and head-to-tail compact hairpin setups, respectively) which is in good agreement with the experimental data.

In total, each of the four molecular systems had an average size of ~90x140x165 Å^3^ including ~170000 atoms (2 apoE monomers, ~200 POPC molecules, ~150 Na^+^ and Cl^-^ ions, and ~48500 water molecules; S3 Fig). All molecular dynamics calculations were performed in the isothermal-isobaric ensembles at 300 K with the program NAMD2.9 [72]. The CHARMM 27 force-field [73, 74] with CMAP corrections [75] was used for protein, water and ions and a united atom force field [76] described the lipid molecules. Long-range electrostatic interactions were calculated using the particle-mesh Ewald method [93]. A smoothing function was applied to truncate short-range electrostatic interactions. The Verlet-I/r-RESPA multiple time-step propagator [94] was used to integrate the equation of motions using a time step of 2 and 4 fs for short- and long-range forces, respectively. All bonds involving hydrogen atoms were constrained using the Rattle algorithm [95].

The systems were equilibrated in 4 steps. First, a 1 ns equilibration was carried out with proteins and lipids fixed. Secondly, a 2 ns equilibration was performed with proteins released in order to remove bad contacts with the lipids. In this step the respectively satisfied XLs were maintained by imposing a restraint potential with a force constant of 200 kcal/mol to the XL Cα-Cα pairs with an upper bound distance of 30 Å beyond which no potential was applied. Applying the same restraint potential, a third 5-ns equilibration was performed with all atoms released. Fourthly, unrestrained equilibration was carried out for 20 ns. Following the equilibration steps, 75 ns molecular dynamics simulations were performed on each of the models.

**Analysis of the molecular dynamics trajectories.** The Euclidean Cα distance between Lys residues involved in XL (that should be below 30 Å [89]) was measured in VMD [90]. The XLs fulfilled in the initial systems were almost all satisfied at the end of the simulation irrespective of the system (S3 Table). The main exception is the XLs involving the N-terminus Lys1 which showed a higher occurrence to be broken given its flexibility. Secondary structure was calculated for each residue with STRIDE [50]. The solvent-accessible surface area (SASA) of positively charged residues, involved in receptor recognition, was measured with VMD in the different simulations (S5 Fig). Their accessibility was also measured in relevant high-resolution apoE structures for comparison (S5 Fig). Average density profiles of the lipid phase and of the protein were calculated using the ST-analyser suite [96]. A typical profile is shown in S4 Fig.

**References**

83. Dong LM, Wilson C, Wardell MR, Simmons T, Mahley RW, Weisgraber KH, et al. Human apolipoprotein E. Role of arginine 61 in mediating the lipoprotein preferences of the E3 and E4 isoforms. J Biol Chem. 1994; 269(35):22358–65. PMID: 8071364.

84. Dong LM, Parkin S, Trakhanov SD, Rupp B, Simmons T, Arnold KS, et al. Novel mechanism for defective receptor binding of apolipoprotein E2 in type III hyperlipoproteinemia. Nat Struct Biol. 1996; 3(8):718–22. https://doi.org/10.1038/nsb0896-718 MID: 8756331.

85. Segelke BW, Forstner M, Knapp M, Trakhanov SD, Parkin S, Newhouse YM, et al. Conformational flexibility in the apolipoprotein E amino-terminal domain structure determined from three new crystal forms: implications for lipid binding. Protein Sci. 2000; 9(5):886–97. https://doi.org/10.1110/ps.9.5.886 PMID: 10850798.

86. Wang G, Pierens GK, Treleaven WD, Sparrow JT, Cushley RJ. Conformations of human apolipoprotein E(263-286) and E(267-289) in aqueous solutions of sodium dodecyl sulfate by CD and 1H NMR. Biochemistry. 1996; 35(32):10358–66. https://doi.org/10.1021/bi960934t PMID: 8786691.

87. Wilson C, Mau T, Weisgraber KH, Wardell MR, Mahley RW, Agard DA. Salt bridge relay triggers defective LDL receptor binding by a mutant apolipoprotein. Structure. 1994; 2(8):713–18. https://doi.org/10.1016/S0969-2126(00)00072-1 PMID: 7994571.

88. Dong J, Peters-Libeu CA, Weisgraber KH, Segelke BW, Rupp B, Capila I, et al. Interaction of the N-terminal domain of apolipoprotein E4 with heparin. Biochemistry. 2001; 40(9):2826–34. https://doi.org/ 10.1021/bi002417n PMID: 11258893.

89. Leitner A, Walzthoeni T, Kahraman A, Herzog F, Rinner O, Beck M, et al. Probing native protein structures by chemical cross-linking, mass spectrometry, and bioinformatics. Mol Cell Proteomics. 2010; 9(8):1634–49. https://doi.org/10.1074/mcp.R000001-MCP201 PMID: 20360032.

90. Humphrey W, Dalke A, Schulten K. VMD: Visual molecular dynamics. J Mol Graph. 1996; 14(1):33–8. https://doi.org/10.1016/0263-7855(96)00018-5 PMID: 8744570.

91. Jo S, Kim T, Iyer VG, Im W. CHARMM-GUI: A web-based graphical user interface for CHARMM. J Comput Chem. 2008; 29(11):1859–65. https://doi.org/10.1002/jcc.20495 PMID: 18351591.

92. Bas DC, Rogers DM, Jensen JH. Very fast prediction and rationalization of pKa values for protein-ligand complexes. Proteins. 2008; 73(3):765–83. https://doi.org/10.1002/prot.22102 PMID: 18498103.

93. Darden T, York D, Pedersen L. Particle mesh Ewald: An *N*•log(*N*) method for Ewald sums in large systems. J Chem Phys. 1993; 98(12):10089. https://doi.org/10.1063/1.464397

94. Tuckerman M, Berne BJ, Martyna GJ. Reversible multiple time scale molecular dynamics. J Chem Phys. 1992; 97(3):1990. https://doi.org/10.1063/1.463137

95. Andersen HC. Rattle: A “velocity” version of the shake algorithm for molecular dynamics calculations. J Comput Phys. 1983; 52(1):24–34. https://doi.org/10.1016/0021-9991(83)90014-1

96. Jeong JC, Jo S, Wu EL, Qi Y, Monje-Galvan V, Yeom MS, et al. ST-analyzer: A web-based user interface for simulation trajectory analysis. J Comput Chem. 2014; 35(12):957–63. https://doi.org/10.1002/jcc.23584 PMID: 24638223.
