# Supplementary figures and images for: Lipidated apolipoprotein E4 structure and its receptor binding mechanism determined by a combined cross-linking coupled to mass spectrometry and molecular dynamics approach
Source: PLoS Comput Biol. 2018 Jun 22;14(6):e1006165. doi: 10.1371/journal.pcbi.1006165 (PMC6033463; doi:10.1371/journal.pcbi.1006165)

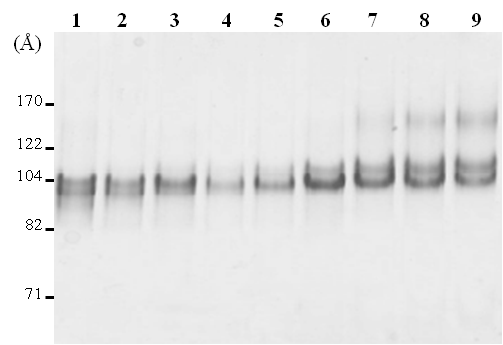

Supplement: S1 Fig — Samples were run on a native PAGE gradient (3.5–13%) and revealed with a Coomassie blue staining. Lanes 1 to 9: apoE4 incubated with 80-, 90-, 100-, 110-, 120-, 130-, 140-, 150-, and 160-fold molar excess of POPC. (TIFF) [file pcbi.1006165.s002.tiff]

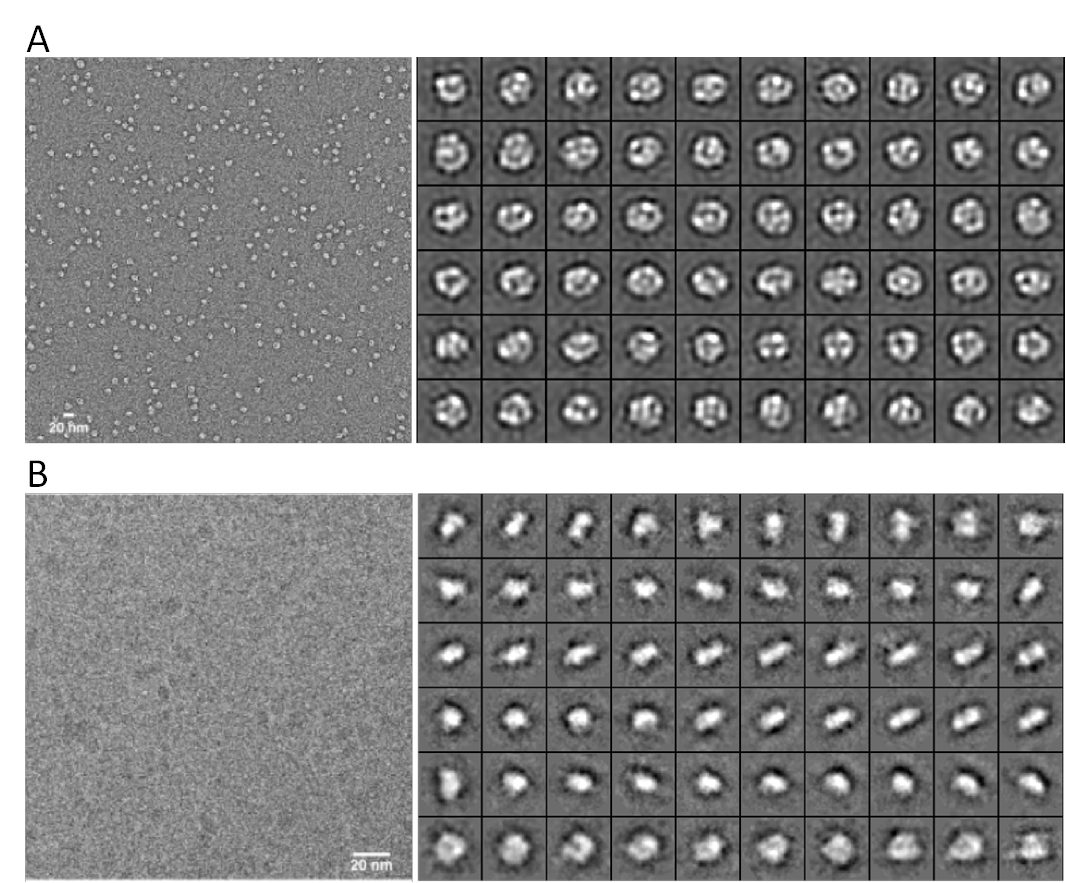

Supplement: S2 Fig — Negative-stain TEM (A) and cryo-TEM (B) images (left) and selected class-averages (right) of the reconstituted apoE4/POPC particles. (TIFF) [file pcbi.1006165.s003.tiff]

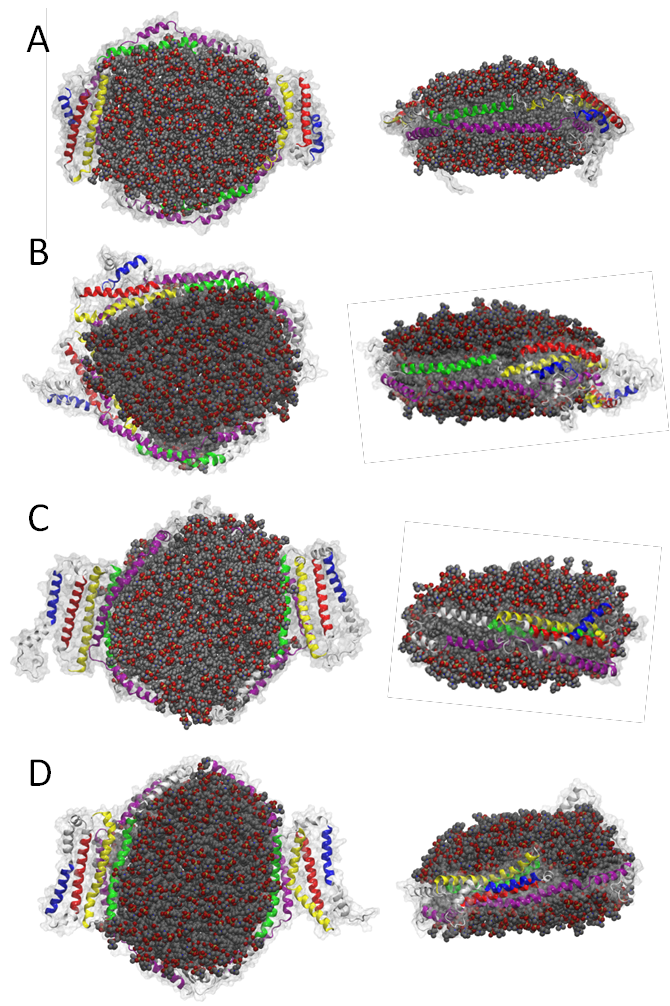

Supplement: S3 Fig — The top (left) and side (right) views of the head-to-tail (A) and head-to-head (B) opened hairpin systems and the head-to-tail (C) and head-to-head (D) compact hairpin systems are depicted. The POPC lipids are shown as van der Waals spheres and the protein as cartoon colored as in Fig 3 have been used. In addition, the protein surface is shown in transparent. (TIFF) [file pcbi.1006165.s004.tiff]

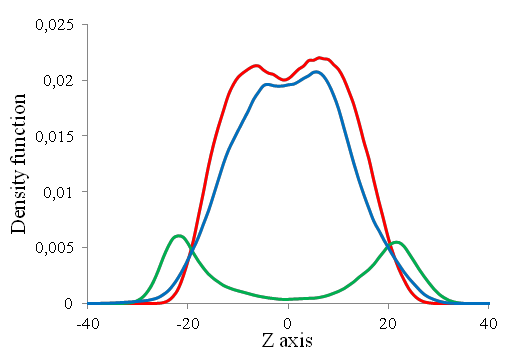

Supplement: S4 Fig — The average density function of the lipid head (green) and tail groups (red), and the protein (blue) is plotted against the principal axis (z) of the system. The average density profile computed for the lipids and the protein showed a stable co-localization of both proteins and lipid acyl chains. These data were extracted from the 75-ns long molecular dynamics trajectory of the head-to-tail opened hairpin system. The curves are representative for all four simulations. (TIFF) [file pcbi.1006165.s005.tiff]

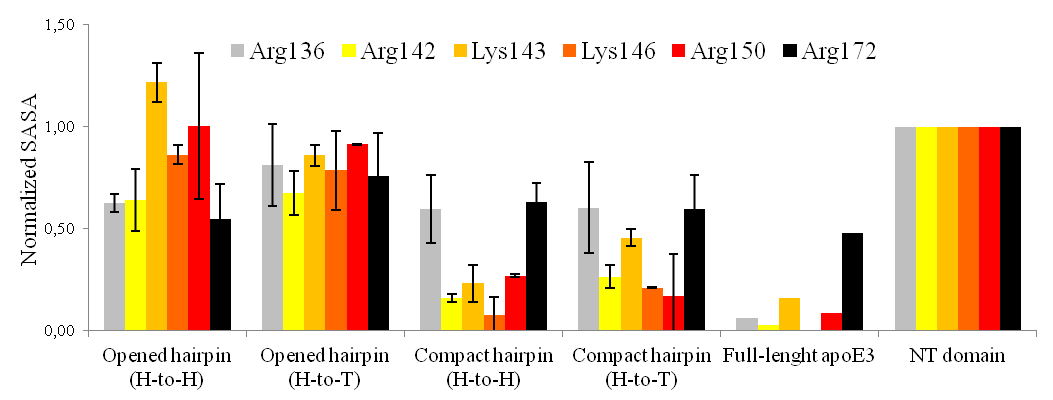

Supplement: S5 Fig — Normalized SASA values of positively charged residues involved in recognition of LDL receptors. Accessibility values are averaged over the two apoE4 molecules present at the surface of the discs. For each configuration (H-to-H, head-to-head; H-to-T, head-to-tail) the SASA was averaged over the 75 ns long molecular dynamics simulations. SASA values were also calculated for the full-length mutated apoE3 structure (PDB code 2L7B) and the apoE3 NT domain (PDB code 2KC3). (TIFF) [file pcbi.1006165.s006.tiff]

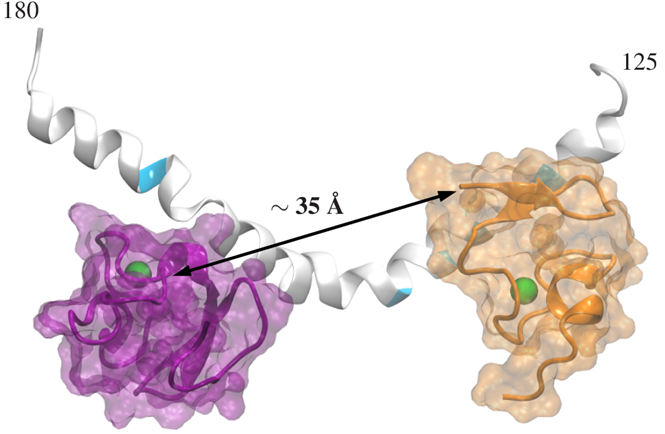

Supplement: S6 Fig — The docked pose of LA4 (purple surface/cartoon) and LA5 repeats (orange surface/cartoon) (PDB code 2LGP) leading to the shortest distance between the two modules is shown. On the apoE4 helix (white cartoon, res. 125–180), residues important for LDLr binding are highlighted in cyan. In addition, the calcium ions complexed by LA4 and LA5 repeats are depicted as green van der Waals spheres. (TIFF) [file pcbi.1006165.s007.tiff]
