# Supplementary material for: Lipidated apolipoprotein E4 structure and its receptor binding mechanism determined by a combined cross-linking coupled to mass spectrometry and molecular dynamics approach
Source: PLoS Comput Biol. 2018 Jun 22;14(6):e1006165. doi: 10.1371/journal.pcbi.1006165 (PMC6033463; doi:10.1371/journal.pcbi.1006165)
Supplement: S1 Table — aAbsolute position of the cross-linked Lys residues and their localization in the secondary structure elements of apoE4 (NTD: N-terminal domain, CTD: C-terminal domain) color-coded as in Fig 3. bExact amino acid sequence of the cross-linked peptides with the cross-linked Lys residues highlighted in red. cxQuestS is a weighted sum of four subscores (xcorrc, xcorrx, match-odds and TIC) that is used to assess the quality of the composite MS2 spectrum as calculated by xQuest. ddeltaS gives the delta score of the respective XL and is a measure for how close the best assigned hit was scored in regard to the second best as calculated by xProphet. (PDF) [file pcbi.1006165.s008.pdf]

Lys 1[a]		Lys 2[a]		Peptide 1 - Peptide 2[b]	*xQuest*S[c]	*delta*S[d]
Lys1	N terminus	Lys95	NTD H3	MKVEQAVETEPEPELR - LSKELQAAQAR	29.33	0
Lys1	N terminus	Lys233	CTD	MKVEQAVETEPEPELR - LDEVKEQVAEVR	21.44	66
Lys1	N terminus	Lys233	CTD	MKVEQAVETEPEPELR - DRLDEVKEQVAEVR	27.46	0
Lys1	N terminus	Lys242	CTD	MKVEQAVETEPEPELR - AKLEEQAQQIR	27.43	69
Lys69	NTD H2	Lys75	NTD H2	ALMDETMKELK - AYKSELEEQLTPVAEETR	22.03	0
Lys69	NTD H2	Lys95	NTD H3	ALMDETMKELK - LSKELQAAQAR	27.15	86
Lys72	NTD H2	Lys95	NTD H3	ELKAYK - LSKELQAAQAR	29.34	55
Lys72	NTD H2	Lys233	CTD	ELKAYK - LDEVKEQVAEVR	27.72	51
				ELKAYK - DRLDEVKEQVAEVR	25.63	33
Lys72	NTD H2	Lys242	CTD	ELKAYK - AKLEEQAQQIR	28.83	47
				ALMDETMKELKAYK - AKLEEQAQQIR	26.09	0
Lys72	NTD H2	Lys282	CTD	ELKAYK - QWAGLVEKVQAAVGTSAAPVPSDNHG	21.91	0
Lys75	NTD H2	Lys95	NTD H3	AYKSELEEQLTPVAEETR - LSKELQAAQAR	26.28	0
Lys75	NTD H2	Lys143	NTD H4	AYKSELEEQLTPVAEETR - KLR	21.31	0
Lys75	NTD H2	Lys242	CTD	AYKSELEEQLTPVAEETR - AKLEEQAQQIR	21.96	0
Lys95	NTD H3	Lys143	NTD H4	LSKELQAAQAR - KLR	28.31	42
Lys95	NTD H3	Lys157	NTD H4	LSKELQAAQAR - DADDLQKR	30.06	67
Lys95	NTD H3	Lys233	CTD	LSKELQAAQAR - LDEVKEQVAEVR	28.43	71
Lys95	NTD H3	Lys242	CTD	LSKELQAAQAR - AKLEEQAQQIR	31.13	62
Lys95	NTD H3	Lys262	CTD	LSKELQAAQAR - LKSWFEPLVEDMQR	19.58	71
Lys95	NTD H3	Lys282	CTD	LSKELQAAQAR - QWAGLVEKVQAAVGTSAAPVPSDNHG	21.30	0
Lys143	NTD H4	Lys233	CTD	KLR - DRLDEVKEQVAEVR	29.51	42
				KLR - LDEVKEQVAEVR	27.45	44

Lys157	NTD H4	Lys233	CTD	DADDLQKR - LDEVKEQVAEVR	33.62	61
Lys157	NTD H4	Lys242	CTD	DADDLQKR - AKLEEQAQQIR	33.89	0
Lys233	CTD	Lys242	CTD	LDEVKEQVAEVR - AKLEEQAQQIR	35.07	64
				DRLDEVKEQVAEVR - AKLEEQAQQIR	29.65	55
