# Supplementary material for: Lipidated apolipoprotein E4 structure and its receptor binding mechanism determined by a combined cross-linking coupled to mass spectrometry and molecular dynamics approach
Source: PLoS Comput Biol. 2018 Jun 22;14(6):e1006165. doi: 10.1371/journal.pcbi.1006165 (PMC6033463; doi:10.1371/journal.pcbi.1006165)
Supplement: S2 Table — aThe constraints were always applied to the Cα-Cα distance.bCα-Cα distances extracted from the NMR structure of soluble apoE3. Distances between residues were measured using VMD and averaged on the different NMR structures available. (PDF) [file pcbi.1006165.s009.pdf]

	Reasoning	Constraints were applied to res.–res.[a]	Distance constraint (in Å)	+ Å	- Å
**Opened hairpin model**					
**A**	Hinge at 186–193 by zipping NT helices 3 and 4 with the CT domain	90–284, 94–281, 98–277, 101–273, 105–270, 112–262, 116–258, 124–250, 126–250, 130–247, 134–243, 138–240, 143–236, 146–233, 153–228, 157–219, 161–215, 164–211, 171–211, 175–208, 179–204, 183–198, 185–194	6	4	4
**B**	NT helix 2 paired with NT helix 3 and outside of the lipid disc	57–110, 65–102, 76–91	6	4	4
**C**	Helices 1 and 2 bundled	26–70[b]	6.25	0.3	0.2
		29–67[b]	5.25	0.5	0.3
		33–63[b]	5.5	0.6	0.7
		36–60[b]	6.5	0.5	0.8
		40–56[b]	6	0.9	1.2
		51–56[b]	9	0.8	0.3
**D**	Discoidal curvature	189–287	105	5	5
		189–235, 235–287	70	5	5
**E**	CT end outside the lipid disc	85–292	6	4	4
**Compact hairpin model**					
**F**	Hinge at 186–193 by zipping NT helix 4 with the CT domain	130–248, 138–241, 142–237, 145–233, 153–226, 157–222, 160–219, 167–211, 175–207, 183–198, 185–194	6	4	4
**G**	NT helix 3 paired with NT helix 4 and NT helix 2 paired with CT domain / NT helices 3 and 2 outside the lipid disc	94–158, 102–150, 113–138, 120–131, 58–242, 62–238, 69–231, 77–226	6	4	4
**H**	Helices 1–3 bundled	26–70[b]	6.25	0.3	0.2
		29–67[b]	5.25	0.5	0.3
		33–63[b]	5.5	0.6	0.7
		36–60[b]	6.5	0.5	0.8
		40–47[b]	9	0.9	1.6
		40–56[b]	6	0.9	1.2
		51–56[b]	9	0.8	0.3
		57–115, 61–112[b]	6.25	0.5	0.5
		64–108[b]	5.25	0.5	0.5
		68–104[b]	8	0.5	0.5
		72–101[b]	9.25	0.5	0.5
		75–97[b]	9.75	0.5	0.5
		79–93[b]	10.5	0.5	0.5
**I**	Discoidal curvature	189–287	105	5	5
		189–235, 235–287	70	5	5
**J**	CT end outside the lipid disc	270–297	6	4	4
