# Supplementary material for: Lipidated apolipoprotein E4 structure and its receptor binding mechanism determined by a combined cross-linking coupled to mass spectrometry and molecular dynamics approach
Source: PLoS Comput Biol. 2018 Jun 22;14(6):e1006165. doi: 10.1371/journal.pcbi.1006165 (PMC6033463; doi:10.1371/journal.pcbi.1006165)
Supplement: S3 Table — a Green check mark: Satisfied XL, Red cross: Non-satisfied XL, -/-: XL not consistent with the specified hairpin model. bXLs are considered to be satisfied if the Euclidean distance between the two Cα atoms is lower than 30 Å in the last 1 ns of the simulation. cFor each configuration (H-to-H, head-to-head; H-to-T, head-to-tail) the fulfillment is given for Monomer1 / Monomer2 (Mon1 / Mon2). (PDF) [file pcbi.1006165.s010.pdf]

XL		Opened hairpin model[b]		Compact hairpin model[b]	
		H-to-H[c]	H-to-T[c]	H-to-H[c]	H-to-T[d]
**Lys 1**	**Lys 2**	**Mon1 / Mon2**	**Mon1 / Mon2**	**Mon1 / Mon2**	**Mon1 / Mon2**
Lys1	Lys95	✓ / ✗	✗ / ✓	✗ / ✓	✓ / ✓
Lys1	Lys233	-/-	-/-	✗ / ✓	✗ / ✓
Lys1	Lys242	-/-	-/-	✗ / ✗	✗ / ✗
Lys69	Lys75	✓ / ✓	✓ / ✓	✓/ ✓	✓ / ✓
Lys69	Lys95	✓ / ✓	✓ / ✓	✓/ ✓	✓ / ✓
Lys72	Lys95	✓ / ✓	✓ / ✓	✓ / ✓	✓ / ✓
Lys72	Lys233	-/-	-/-	✓ / ✓	✓ / ✓
Lys72	Lys242	-/-	-/-	✓ / ✓	✓ / ✓
Lys72	Lys282	✓ / ✓	✓ / ✓	-/-	-/-
Lys75	Lys95	✓ / ✓	✓ / ✓	✓ / ✓	✓ / ✓
Lys75	Lys143	-/-	-/-	✓ / ✓	✓ / ✓
Lys75	Lys242	-/-	-/-	✓ / ✓	✓ / ✓
Lys95	Lys143	-/-	-/-	✓ / ✓	✓ / ✓
Lys95	Lys157	-/-	-/-	✓ / ✓	✓ / ✓
Lys95	Lys233	-/-	-/-	✓ / ✓	✓ / ✓
Lys95	Lys242	-/-	-/-	✓ / ✓	✓ / ✓
Lys95	Lys262	✗ / ✗	✗ / ✗	-/-	-/-
Lys95	Lys282	✓ / ✓	✓ / ✓	-/-	-/-
Lys143	Lys233	✓ / ✓	✓ / ✓	✓ / ✓	✓ / ✓
Lys157	Lys233	✓ / ✓	✓ / ✓	✓ / ✓	✓ / ✓
Lys157	Lys242	✗ / ✓	✓ / ✗	✓ / ✓	✓ / ✓
Lys233	Lys242	✓ / ✓	✓ / ✓	✓ / ✓	✓ / ✓
