# Supplementary material for: Lipidated apolipoprotein E4 structure and its receptor binding mechanism determined by a combined cross-linking coupled to mass spectrometry and molecular dynamics approach
Source: PLoS Comput Biol. 2018 Jun 22;14(6):e1006165. doi: 10.1371/journal.pcbi.1006165 (PMC6033463; doi:10.1371/journal.pcbi.1006165)
Supplement: S4 Table — For each constraint, the atoms of apoE4 and LA5/LA4 repeats are indicated. In all cases, the pair of atoms constrained should form a salt bridge and thus, the distance range was set to 2–5 Å with an optimal distance of 3 Å for each constrained. Constraints between apoE4 and LA4/LA5 repeats were treated ambiguously/unambiguously. (PDF) [file pcbi.1006165.s011.pdf]

**ApoE4 (125–185)**	**LA5**	**LA4**
R136 C_ζ	E180 C_δ	
K143 N_ε	E187 C_δ	
K146 N_ε	D196 C_γ	
K146 N_ε	D200 C_γ	
R172 C_ζ		E147 C_δ
R172 C_ζ		E149 C_δ
R172 C_ζ		E151 C_δ
